# MomsTalkShots, tailored educational app, improves vaccine attitudes: a randomized controlled trial

**DOI:** 10.1186/s12889-022-14498-7

**Published:** 2022-11-21

**Authors:** Matthew Z. Dudley, Saad B. Omer, Sean T. O’Leary, Rupali J. Limaye, Mallory K. Ellingson, Christine I. Spina, Sarah E. Brewer, Robert A. Bednarczyk, Allison T. Chamberlain, Fauzia Malik, Paula M. Frew, Cathy Church-Balin, Laura E. Riley, Kevin A. Ault, Walter A. Orenstein, Neal A. Halsey, Daniel A. Salmon

**Affiliations:** 1 Department of International Health, Johns Hopkins University Bloomberg School of Public Health, 615 N Wolfe St, W5041, Baltimore, MD 21205 USA; 2 Institute for Vaccine Safety, Johns Hopkins University Bloomberg School of Public Health, 615 N. Wolfe Street, Baltimore, MD 21205 USA; 3 Yale Institute for Global Health, Yale School of Medicine, New Haven, USA; 4 Department of Infectious Diseases, Yale School of Medicine, New Haven, USA; 5 Department of Epidemiology of Microbial Diseases, Yale School of Public Health, New Haven, USA; 6 Department of Health Policy and Management, Yale School of Public Health, New Haven, USA; 7 Adult and Child Center for Health Outcomes Research and Delivery Science, University of Colorado Anschutz Medical Campus and Children’s Hospital Colorado, 1890 N Revere Ct. Mailstop F443, Aurora, CO 80045 USA; 8 Department of Pediatrics, University of Colorado Anschutz Medical Campus, 13123 E 16th Ave, Aurora, CO 80045 USA; 9 Department of Health, Behavior and Society, Johns Hopkins University Bloomberg School of Public Health, 615 N. Wolfe Street, Baltimore, MD 21205 USA; 10 Department of Epidemiology, Johns Hopkins University Bloomberg School of Public Health, 615 N. Wolfe Street, Baltimore, MD 21205 USA; 11 International Vaccine Access Center, Johns Hopkins University Bloomberg School of Public Health, 615 N. Wolfe Street, Baltimore, MD 21205 USA; 12 Department of Family Medicine, University of Colorado Anschutz Medical Campus, 13001 E 17th Pl, Aurora, CO 80045 USA; 13 Hubert Department of Global Health, Rollins School of Public Health, Emory University, 1518 Clifton Rd. NE, Atlanta, GA 30322 USA; 14 Department of Epidemiology, Rollins School of Public Health, Emory University, 1518 Clifton Rd. NE, Atlanta, GA 30322 USA; 15 Emory Vaccine Center, Emory University, 201 Dowman Drive, Atlanta, GA 30322 USA; 16 Department of Health Policy and Management, Yale School of Public Health, 60 College Street, New Haven, CT 06520-0834 USA; 17 Division of Infectious Diseases, Department of Medicine, School of Medicine, Emory University, 2015 Uppergate Dr, Atlanta, GA 30322 USA; 18 School of Public Health; School of Medicine; Population Health & Health Equity Initiative, Office of Research and Economic Development, University of Nevada, 4505 S. Maryland Pkwy, Las Vegas, NV 89154 USA; 19 Present address: Merck & Co., Inc., (at Emory University and the University of Nevada – not Merck – when work was performed), NJ Kenilworth, USA; 20 Center for Communication Programs, Johns Hopkins University Bloomberg School of Public Health, 615 N. Wolfe Street, Baltimore, MD 21205 USA; 21 Department of Obstetrics and Gynecology, Weill Cornell Medicine, 525 East 68th Street, New York, NY 10065 USA; 22 Department of Obstetrics and Gynecology, University of Kansas School of Medicine, 3901 Rainbow Boulevard, Kansas City, KS 66160 USA; 23 Department of Pediatrics, School of Medicine, Emory University, 2015 Uppergate Dr, Atlanta, GA 30322 USA

**Keywords:** Vaccines, Pregnancy, Knowledge, Attitudes, Beliefs, App, Education, Intentions, Trust, Hesitancy

## Abstract

**Background:**

Many pregnant women and parents have concerns about vaccines. This analysis examined the impact of MomsTalkShots, an individually tailored educational application, on vaccine attitudes of pregnant women and mothers.

**Methods:**

MomsTalkShots was the patient-level component of a multi-level intervention to improve maternal and infant vaccine uptake that also included provider- and practice-level interventions. The impact of these interventions was studied using a two-by-two factorial design, randomizing at both the patient- and the practice-level. Study staff recruited pregnant women from a diverse set of prenatal care practices in Colorado and Georgia between June 2017 and July 2018. All participants (*n* = 2087) received a baseline survey of maternal and infant vaccine intentions and attitudes, and two follow-up surveys at least 1 month and 1 year after their infant’s birth, respectively. Half of participants (*n* = 1041) were randomly assigned to receive educational videos through MomsTalkShots, algorithmically tailored to their vaccine intentions, attitudes, and demographics. Since the practice/provider intervention did not appear impactful, this analysis focused on MomsTalkShots regardless of the practice/provider intervention.

**Results:**

By 1 month post-birth, MomsTalkShots increased perceived risk of maternal influenza disease (61% among MomsTalkShots recipients vs 55% among controls; Odds Ratio: 1.61, 95% Confidence Interval: 1.23–2.09), confidence in influenza vaccine efficacy (73% vs 63%; OR: 1.97, 95%CI: 1.47–2.65), and perceived vaccine knowledge (55% vs 48%; OR: 1.39, 95%CI: 1.13–1.72). Among those intending not to vaccinate at baseline, MomsTalkShots increased perceived risk of maternal influenza disease (38% vs 32%; OR: 2.07, 95%CI: 1.15–3.71) and confidence in influenza vaccine efficacy (44% vs 28%; OR: 2.62, 95%CI: 1.46–4.69).

By 1 year post-birth, MomsTalkShots increased perceived vaccine knowledge (62% vs 50%; OR: 1.74, 95%CI: 1.36–2.24) and trust in vaccine information from obstetricians and pediatricians (64% vs 55%; OR: 1.53, 95%CI: 1.17–2.00). Among those uncertain about vaccinating at baseline, MomsTalkShots increased perceived vaccine knowledge (47% vs 12%; OR: 6.89, 95%CI: 1.52–31.25) and reduced infant vaccine safety concerns (71% vs 91%; OR: 0.24, 95%CI: 0.06–0.98).

**Conclusions:**

MomsTalkShots improved pregnant women’s and mothers’ knowledge and perceptions of maternal and infant vaccines and the diseases they prevent, and offers a scalable tool to address vaccine hesitancy.

**Trial registration:**

Registered at Clinicaltrials.gov on 13/09/2016 (registration number: NCT02898688).

**Supplementary Information:**

The online version contains supplementary material available at 10.1186/s12889-022-14498-7.

## Background

Only 55% of pregnant women in the United States (US) received influenza vaccination during the 2020–2021 influenza season [1]. Though this exceeded the 50% coverage among all adults, [2] it fell well short of the Healthy People 2020 goal of 80% coverage among pregnant women [3]. Only 54% received tetanus, diphtheria, and acellular pertussis (Tdap) vaccination [1].

Many pregnant women [4–11] and parents [12–28] have concerns about vaccines. Vaccine knowledge, attitudes and beliefs (KABs) are strongly associated with vaccine behavior [29]. Parents often start making vaccine decisions for their children during or soon after their first pregnancy, especially those with negative attitudes toward vaccines [30]. The prenatal period is thus an optimal time to educate about both maternal and infant vaccines, potentially reaching an undecided audience before overexposure to misinformation leads to rigid misperceptions [31].

However, many pregnant women do not receive adequate information about vaccines directly from their prenatal care providers, instead relying on internet searches and their social networks [11]. Healthcare providers are busy, and discussions with vaccine hesitant patients can be long and burdensome [32]. Many prenatal care providers do not have the same level of experience and training discussing vaccination with their patients as pediatricians [33]. Tools are needed to address maternal and infant vaccine concerns and ease this burden from providers, but messaging must be tailored, [34, 35] as vaccine KABs and acceptance have been shown to differ substantially by gender, education, socioeconomic status, ethnicity and race, [36–45] including among pregnant women [46].

As one component of a multi-level intervention to encourage immunization of pregnant women and their infants, [47, 48] we designed MomsTalkShots, an individually tailored educational application (app) [49]. Among a sample of pregnant women from Georgia and Colorado, MomsTalkShots substantially increased influenza vaccine uptake among women who initially did not intend to vaccinate during pregnancy. No significant increase in Tdap vaccine uptake was found though baseline Tdap vaccine coverage was very high across all practices [48]. The main objective of this analysis was to evaluate the impact of MomsTalkShots on vaccine KABs.

## Materials and methods

### MomsTalkShots

The MomsTalkShots app (described in more detail elsewhere) [49] was designed as a website to be accessible via multiple internet browsers on smartphones, tablets, and computers. The app begins with registration, then administers a survey, then immediately after survey completion provides educational videos that are algorithmically responsive to its users’ vaccine intentions, KABs, and demographics [49]. The videos incorporate introductions and conclusions from obstetricians and pediatricians of different races/ethnicities with narrated animation to communicate messages in an interesting and engaging manner. The videos were designed based on approaches shown to be effective in training healthcare providers to improve their vaccine discussions with patients: for patients already intending to vaccinate, taking a presumptive approach; and for patients with concerns, establishing empathy, then carefully addressing the concerns within the context of the risk of disease, the benefits of vaccination, and the ability to protect through vaccinating [50–54]. Videos covered both maternal and infant vaccination, though videos provided to pregnant women focused more on the former and videos provided to new mothers focused more on the latter. Videos were also available in a gallery for rewatching upon logging in between survey timepoints. A feature allowing pregnant women to refer the app to their close contacts was evaluated elsewhere and was shown to increase influenza vaccine uptake among family and friends to cocoon the infant [55, 56].

### Study context

MomsTalkShots was the patient-level component of a multi-level intervention to improve maternal and infant vaccine uptake that also included provider- and practice-level interventions, described in more detail elsewhere [47, 48]. The impact of these interventions was studied using a two-by-two factorial design, randomizing at both the patient- and the practice-level (provider-level interventions were implemented and analysed in tandem with practice-level interventions). The practice-level intervention implemented an adaptation of the Centers for Disease Control and Prevention (CDC) Assessment, Feedback, Incentives and Exchange (AFIX) model, [57] and the provider-level interventions included provision of the Continuing Medical Education (CME) module *VaxChat* [58] and the book *The Clinician’s Vaccine Safety Resource Guide* [50]. The target sample size for the overall trial of 1896 was calculated using PASS 11 (NCSS, LLC, Kaysville UT), assuming a cluster randomized design with intra-class correlation coefficient of 0.01 (based on preliminary data from a previous pilot study), [59] to provide a power of 80% (α = 0.05) to see a doubling in maternal Tdap vaccinations (not for the purpose of this analysis) [48].

### Recruitment

Study staff recruited pregnant women from waiting rooms of a geographically and socio-demographically diverse set of prenatal care practices in Colorado and Georgia between June 2017 and July 2018 and followed them through March 2019. Practices were chosen to capture diversity in patient demographics, urbanicity, provider types, and practice size. Eleven of the 22 participating practices were assigned to receive the practice- and provider-level interventions (via covariate constrained cluster randomization) [60]. Patient eligibility criteria included: between 18 and 50 years of age, English-speaking, and gestational age of 8–26 weeks (since Tdap is recommended at 27–36 weeks gestation) [61]. Upon enrollment, study staff lent participants an electronic tablet to complete the baseline survey via the app while in the waiting room. Two follow-up surveys were made available to all participants via the app approximately 1 month and 1 year after their infant’s birth, respectively, to complete using their device of choice at home. A $20 incentive was provided for completion of each survey. Further detail and data on recruitment is reported elsewhere [48].

### Randomization

All participants received three surveys, but only the half randomly assigned to receive the patient-level intervention subsequently received the tailored educational videos (MomsTalkShots) immediately after the baseline and initial follow-up surveys. We used SAS PROC PLAN to generate patient-level randomization schedules for each of the 22 participating practices, using block randomization with a block size of 8; these randomization sequences were then programmed into the app such that participants were randomized upon registration, blinded to study investigators.

### Data collection

Surveys used multiple choice questions to assess vaccine intentions, and Likert scale statements to assess latent vaccine KAB constructs, including: confidence in vaccine safety and efficacy, perceived susceptibility to and severity of vaccine-preventable diseases, self-efficacy (one’s belief in their ability to execute behaviors necessary to reach specific goals), [62] descriptive (what people do) and injunctive (what people approve) norms, [63] perceived vaccine knowledge, and trust in sources of vaccine information. These constructs were chosen after review of relevant behavioral models and scales, [8, 64] and multiple survey items were dedicated to each construct. Likert scale response options included: strongly agree, agree, disagree, and strongly disagree; knowledge and trust statements also included “don’t know”; and trust statements regarding pediatricians and naturopathic/chiropractic doctors also included “I don’t have a pediatrician yet” and “I don’t see this type of doctor,” respectively. Specific vaccine safety concern statements were administered only to those who expressed a lack of confidence in vaccine safety (to avoid creating new concerns).

### Data analysis

Likert scale responses to survey items were encoded as follows: 1 - strongly disagree; 2 - disagree; 3 - don’t know; 4 - agree; 5 - strongly agree. Those who agreed (or strongly agreed) with survey items expressing confidence in vaccine safety were encoded to have disagreed (or strongly disagreed) with survey items expressing specific vaccine safety concerns. Those who had not (yet) seen pediatricians and/or naturopathic/chiropractic doctors were treated as missing for these statements.

Summary scores for constructs combined the scores from their constituent statements. Only constructs with multiple statements assessed at two or more timepoints were analysed. All scores were standardized by dividing into the maximum score then multiplying by 100. This standardization allowed comparison of construct summary scores with different numbers of contributing statements.

Dichotomous variables were encoded for each of the survey items as follows: 1 - agree or strongly agree; 0 - disagree, strongly disagree, or don’t know. Dichotomous construct summary scores were created by calculating the mean of each scaled continuous construct summary score and encoding as above the mean (“high”) or below the mean (“low”).

Analysis was guided by the statistical analysis plan documented in the study protocol and recorded on clinicaltrials.gov. Multilevel mixed-effects linear regressions were performed for scaled statement and construct scores, with a random intercept for the clinic. Interim and final time point scores were each regressed on their corresponding baseline score, to control for potential differences in baseline scores between groups. Dummy variables were initially included for MomsTalkShots, the practice/provider intervention, and their product (to test for potential interaction). However, the interaction term was found not to be statistically significant, so the model was reparametrized without a dummy variable for interaction. Regressions were stratified by baseline intention to vaccinate. Dichotomous/logistic analysis otherwise emulating the above methodology was performed to increase interpretability. Statistical significance was considered *p* < 0.05.

### Data presentation

Per protocol, because our surveys included so many individual items, we focused our analysis on constructs, to reduce redundancy and the chances of type 1 error. However, individual survey statements were also analysed, to provide additional insight into the individual components driving changes in constructs. The analyses of individual survey statements are reported as appendices.

Since the practice/provider intervention did not appear impactful, and the impact of MomsTalkShots did not significantly differ between intervention and non-intervention practices, we focused on MomsTalkShots regardless of the practice/provider intervention.

The results of the dichotomous analysis (Additional file 1: Appendices 1–2) were highly consistent with the results of the continuous analysis (Additional file 1: Appendices 3–4), aside from small differences in significance due to loss of power, so we present the results of the dichotomous analysis for greater interpretability.

## Results

### Enrollment and follow-up

Of the 3904 pregnant women found to be eligible after screening, 1391 declined to participate, and 303 did not finish enrollment. Ultimately 2087 enrolled pregnant women were randomized and provided enough data to contribute to this analysis. Informed consent was obtained from all participants.

Nearly three quarters of the participating pregnant women (*n* = 1524; 73%) completed the initial follow-up survey (at least 1 month after their infant’s birth), and over half (*n* = 1117; 54%) completed the final follow-up survey (at least 1 year after their infant’s birth) (Additional file 1: Appendix 7). Follow-up survey completion did not differ by intervention group, but was consistently lower among women not intending to vaccinate at baseline (Additional file 1: Appendix 8).

### Sociodemographic characteristics

Half of participating pregnant women were from Georgia, and half from Colorado. Nearly half (46%) were pregnant for the first time. About 85% provided their race/ethnicity and 83% their education: of these, 64% were White, 16% were Black and 11% were Hispanic, and 72% had at least an undergraduate degree, respectively. Sociodemographic characteristics were similar between study arms, [48] and are described in more detail elsewhere [46, 48, 65].

### Baseline vaccine intentions

Over half (56%) of women intended to receive both influenza and Tdap vaccines during pregnancy, 16% intended to receive one but not the other, 14% intended to receive neither, and 13% were unsure; 81% intended for their baby to receive all recommended vaccines, 11% intended for their baby to receive some or no recommended vaccines, and 8% were unsure (Table 1). Baseline vaccine intentions were similar between study arms, [48] and are described in more detail elsewhere [46, 48, 65].

**Table 1:** Maternal and infant vaccine intentions at baseline stratified by patient intervention (MomsTalkShots) versus patient control

	MomsTalkShots	Control	Total
**Maternal Vaccine Intentions**^a^			
Influenza and Tdap	601 (57)	578 (56)	1179 (56)
Influenza not Tdap	68 (7)	81 (8)	149 (7)
Tdap not Influenza	90 (9)	101 (10)	191 (9)
Neither	156 (15)	146 (14)	302 (14)
Unsure	131 (13)	135 (13)	266 (13)
Total	1046 (100)	1041 (100)	2087 (100)
*P*-value^b^	0.628		
**Infant Vaccine Intentions**^a^			
All On Time	717 (69)	708 (68)	1425 (68)
All But Delayed	120 (11)	140 (13)	260 (12)
Some But On Time	64 (6)	52 (5)	116 (6)
Some But Delayed	32 (3)	31 (3)	63 (3)
None	22 (2)	20 (2)	42 (2)
Unsure	89 (9)	88 (8)	177 (8)
Total	1044 (100)	1039 (100)	2083 (100)
*P*-value^b^	0.709		

^a^Baseline survey questions assessing maternal and infant vaccine intentions, respectively, were: “Current guidelines suggest pregnant women to receive two vaccines while pregnant, flu and whooping cough. I intend to get: 1) both flu and whooping cough vaccines; 2) flu but not whooping cough vaccine; 3) whooping cough but not flu vaccine; 4) no vaccines; 5) not sure” and “Current guidelines suggest babies receive several vaccines. Regarding the vaccinations my doctor recommends for my baby after birth, I intend to get my baby: 1) all recommended vaccines on time; 2) all recommended vaccines but some spread out past the recommended ages; 3) some recommended vaccines but each on time; 4) some recommended vaccines spread out past the recommended ages; 5) no vaccines; 6) I’m not sure yet”

^b^*P*-value for the Pearson chi-squared proportion test at significance level of (a) 5%; bolded if significant

Herein we report statistically significant results among all participants and stratified by vaccine intent.

### Effect of MomsTalkShots among all participants

One month after their infant’s birth, 61% of MomsTalkShots recipients had high perceived risk of maternal influenza disease, compared to 55% of controls (Odds Ratio: 1.61, 95% Confidence Interval: 1.23–2.09); 73% had high confidence in maternal influenza vaccine efficacy, compared to 63% of controls (OR: 1.97, 95%CI: 1.47–2.65); and 55% had high perceived vaccine knowledge, compared to 48% of controls (OR: 1.39, 95%CI: 1.13–1.72) (Table 2). One year after their infant’s birth, 62% had high perceived vaccine knowledge, compared to 50% of controls (OR: 1.74, 95%CI: 1.36–2.24); and 64% had high trust in vaccine information from obstetricians and pediatricians, compared to 55% of controls (OR: 1.53, 95%CI: 1.17–2.00) (Table 3).

**Table 2:** Impact of MomsTalkShots on vaccine KAB constructs of women one month after their infant’s birth stratified by baseline vaccine intentions^a^, dichotomous analysis^b^

	% with above average construct scores^c^ among those not receiving MomsTalkShots				% with above average construct scores^c^ among those receiving MomsTalkShots				Effect of MomsTalkShots on % with above average construct scores^c^, OR (95% CI)^b^			
*Intentions to Vaccinate*^*a*^	*All*	*Yes*	*No*	*Unsure*	*All*	*Yes*	*No*	*Unsure*	*All*	*Yes*	*No*	*Unsure*
**KAB Constructs** ^c^												
Specific safety concerns (for infant vaccines)^fh^	56	49	90	89	53	47	87	81	0.89 (0.70–1.12)	0.94 (0.73–1.20)	0.62 (0.19–2.05)	0.44 (0.14–1.40)
Perceived risk (maternal influenza)^d^	55	64	32	42	61	71	38	44	**1.61 (1.23–2.09)**	**1.57 (1.12–2.20)**	**2.07 (1.15–3.71)**	1.53 (0.70–3.34)
Confidence in vaccine efficacy (maternal influenza vaccine)^d^	63	77	28	39	73	86	44	52	**1.97 (1.47–2.65)**	**2.09 (1.36–3.22)**	**2.62 (1.46–4.69)**	1.96 (0.90–4.26)
Perceived risk (infant whooping cough)^f^	42	47	22	19	39	42	19	31	**0.75 (0.57–1.00)**^**g**^	**0.70 (0.51–0.96)**	0.77 (0.27–2.24)	2.18 (0.68–6.96)
Confidence in vaccine efficacy (whooping cough vaccine)^e^	56	68	24	47	61	72	37	41	1.01 (0.77–1.33)	0.96 (0.69–1.34)	1.42 (0.71–2.86)	0.53 (0.18–1.52
Pro-vaccine social norms^f^	44	49	19	15	46	51	15	33	1.08 (0.86–1.35)	1.05 (0.83–1.34)	0.55 (0.20–1.47)	2.57 (0.97–6.85)
Perceived vaccine knowledge^f^	48	53	24	28	55	59	38	33	**1.39 (1.13–1.72)**	**1.36 (1.08–1.70)**	2.04 (0.97–4.28)	1.00 (0.41–2.45)
Trust in vaccine information (from obstetricians and pediatricians)^f^	53	60	23	20	59	27	26	27	1.22 (0.97–1.54)	1.22 (0.95–1.56)	1.43 (0.63–3.27)	1.12 (0.43–2.93)
Trust in vaccine information (from naturopaths and chiropractors)^f^	56	57	60	51	60	61	59	51	0.83 (0.57–1.19)	0.76 (0.50–1.16)	1.04 (0.33–3.30)	0.83 (0.26–2.61)
Trust in vaccine information (from federal agencies and academic institutions)^f^	48	55	18	13	49	55	14	25	1.06 (0.84–1.34)	1.02 (0.79–1.31)	0.77 (0.29–2.06)	1.88 (0.65–5.47)

^a^For constructs specific to maternal influenza disease or vaccine, “intend to vaccinate”, “intend not to vaccinate”, and “uncertain intentions” refer to maternal influenza vaccine^d^; for constructs specific to maternal pertussis disease or vaccine, “intend to vaccinate”, “intend not to vaccinate”, and “uncertain intentions” refer to maternal Tdap vaccine^e^; for constructs specific to infant diseases or vaccines, “intend to vaccinate”, “intend not to vaccinate”, and “uncertain intentions” refer to intending to receive all recommended infant vaccines versus intending to receive some or no recommended infant vaccines^f^; for constructs relevant to both maternal and infant vaccines, “intend to vaccinate”, “intend not to vaccinate”, and “uncertain intentions” refer to intending to receive all recommended infant vaccines versus intending to receive some or no recommended infant vaccines^f^

^b^OR = Odds Ratio from logistic regression comparing the proportions with above average construct scores^c^ at follow-up between those receiving MomsTalkShots and those not receiving MomsTalkShots, controlling for the corresponding proportions with above average construct scores^c^ at baseline; 95%CI = 95% Confidence Interval; bolded if statistically significant (in the dichotomous analysis)

^c^KAB = knowledge, attitudes, and beliefs. Summary scores were created for all KAB constructs with multiple constituent survey statements at each timepoint (see Additional file 1: Appendix 1). Dichotomous variables assessing construct summary scores coded scores above the average as 1 and scores below the average as 0

^g^This appearance of an overlap with 1 in the 95%CI is due to rounding

^h^The KAB construct “Specific safety concerns (for infant vaccines)” was created from negatively phrased survey statements, so a negative association would indicate a positive effect on vaccine perceptions. All other KAB constructs in this table were created from positively phrased survey statements, so a positive association would indicate a positive effect on vaccine perceptions

**Table 3:** Impact of MomsTalkShots on vaccine KAB constructs of women one year after their infant’s birth stratified by baseline vaccine intentions^a^, dichotomous analysis^b^

	% with above average construct scores^c^ among those not receiving MomsTalkShots				% with above average construct scores^c^ among those receiving MomsTalkShots				Effect of MomsTalkShots on % with above average construct scores^c^, OR (95% CI)^b^			
*Intentions to Vaccinate*^*a*^	*All*	*Yes*	*No*	*Unsure*	*All*	*Yes*	*No*	*Unsure*	*All*	*Yes*	*No*	*Unsure*
**KAB Constructs**^**c**^												
Specific safety concerns (for infant vaccines)^d^	47	39	83	91	44	38	93	71	0.88 (0.67–1.15)	0.93 (0.70–1.25)	2.78 (0.67–11.46)	**0.24 (0.06–0.98)**
Perceived risk (infant whooping cough)	46	49	26	24	39	40	19	44	0.89 (0.66–1.21)	0.89 (0.64–1.22)	0.53 (0.13–2.27)	1.05 (0.27–4.08)
Pro-vaccine social norms	49	54	22	21	55	59	21	38	1.23 (0.95–1.58)	1.22 (0.93–1.61)	0.67 (0.23–1.94)	2.13 (0.70–6.53)
Perceived vaccine knowledge	50	55	29	12	62	65	40	47	**1.74 (1.36–2.24)**	**1.60 (1.22–2.10)**	1.71 (0.73–4.01)	**6.89 (1.52–31.25)**
Trust in vaccine information (from obstetricians and pediatricians)	55	63	16	15	64	70	14	41	**1.53 (1.17–2.00)**	**1.47 (1.09–1.97)**	1.07 (0.32–3.55)	**3.41 (1.03–11.29)**
Trust in vaccine information (from naturopaths and chiropractors)	53	50	62	65	56	56	57	59	1.04 (0.68–1.60)	1.06 (0.66–1.72)	0.89 (0.22–3.62)	0.90 (0.18–4.63)
Trust in vaccine information (from federal agencies and academic institutions)	51	58	14	12	54	59	12	32	1.30 (0.99–1.71)	1.21 (0.90–1.62)	0.93 (0.27–3.24)	3.03 (0.82–11.12)

^a^All constructs in this table are either relevant to both maternal and infant vaccines or specific to infant diseases or vaccines, so intentions to vaccinate refer to intending to receive all recommended infant vaccines versus intending to receive some or no recommended infant vaccines

^b^OR = Odds Ratio from logistic regression comparing the proportions with above average construct scores^c^ at follow-up between those receiving MomsTalkShots and those not receiving MomsTalkShots, controlling for the corresponding proportions with above average construct scores^c^ at baseline; 95%CI = 95% Confidence Interval; bolded if statistically significant (in the dichotomous analysis)

^c^KAB = knowledge, attitudes, and beliefs. Summary scores were created for all KAB constructs with multiple constituent survey statements at each timepoint (see Additional file 1: Appendix 2). Dichotomous variables assessing construct summary scores coded scores above the average as 1 and scores below the average as 0. Constructs assessing KAB about maternal disease and vaccines were not assessed in the final survey one year after birth

^d^The KAB construct “Specific safety concerns (for infant vaccines)” was created from negatively phrased survey statements, so a negative association would indicate a positive effect on vaccine perceptions. All other KAB constructs in this table were created from positively phrased survey statements, so a positive association would indicate a positive effect on vaccine perceptions

### Effect of MomsTalkShots among participants intending to vaccinate

One month after their infant’s birth, 71% of MomsTalkShots recipients intending to vaccinate at baseline had high perceived risk of maternal influenza disease, compared to 64% of controls (OR: 1.57, 95%CI: 1.12–2.20); 86% had high confidence in maternal influenza vaccine efficacy, compared to 77% of controls (OR: 2.09, 95%CI: 1.36–3.22); and 59% had high perceived vaccine knowledge, compared to 53% of controls (OR: 1.36, 95%CI: 1.08–1.70) (Table 2). One year after their infant’s birth, 65% had high perceived vaccine knowledge, compared to 55% of controls (OR: 1.60, 95%CI: 1.22–2.10); and 70% had high trust in vaccine information from obstetricians and pediatricians, compared to 63% of controls (OR: 1.47, 95%CI: 1.09–1.97) (Table 3).

### Effect of MomsTalkShots among participants intending not to vaccinate

One month after their infant’s birth, 38% of MomsTalkShots recipients intending not to vaccinate at baseline had high perceived risk of maternal influenza disease, compared to 32% of controls (OR: 2.07, 95%CI: 1.15–3.71); and 44% had high confidence in maternal influenza vaccine efficacy, compared to 28% of controls (OR: 2.62, 95%CI: 1.46–4.69) (Table 2).

### Effect of MomsTalkShots among participants with uncertain vaccine intentions

One year after their infant’s birth, 47% of MomsTalkShots recipients with uncertain vaccine intentions at baseline had high perceived vaccine knowledge, compared to 12% of controls (OR: 6.89, 95%CI: 1.52–31.25); and 71% had high specific safety concerns for infant vaccines, compared to 91% of controls (OR: 0.24, 95%CI: 0.06–0.98) (Table 3).

### Potential unintended effects

MomsTalkShots decreased perceived risk of infant pertussis: 1 month after their infant’s birth, 39% of MomsTalkShots recipients had high perceived risk of infant pertussis, compared to 42% of controls (OR: 0.75, 95%CI: 0.57–1.00) (Table 2). While MomsTalkShots decreased perceived susceptibility; it also increased perceived severity (Additional file 1: Appendix 1), and the reduction in perceived susceptibility was limited to those who received Tdap during pregnancy (Additional file 1: Appendix 5).

## Discussion

MomsTalkShots positively impacted pregnant women’s and mothers’ knowledge and perceptions of maternal and infant vaccines and the diseases they prevent. Among women initially intending not to vaccinate, MomsTalkShots increased perceived risk of maternal influenza disease and confidence in influenza vaccine efficacy. Among women with uncertain infant vaccine intentions, MomsTalkShots increased trust in obstetricians and pediatricians and substantially reduced safety concerns.

These findings correspond to our previous findings that MomsTalkShots substantially increased influenza vaccine uptake among pregnant women who initially did not intend to vaccinate during pregnancy [48]. This contributes to the literature showing that changing attitudes can improve vaccine acceptance [29, 35]. These findings are notable, as vaccine education is typically ineffective unless implemented in tandem with other proven interventions (such as provider prompts and standing orders), [31, 66] with a few exceptions [67–71]. MomsTalkShots’ impact may be partly due to its ability to tailor its content to individual vaccine intentions, KABs, and demographics, further supporting such an approach to vaccine education [34, 35]. Also notable is the duration of impact; decreased safety concerns, increased perceived vaccine knowledge, and increased trust in obstetricians and pediatricians were found in women nearly a year after they received MomsTalkShots, despite evidence that didactic education often fades from memory after about a week [72].

The only unexpected construct association found was that MomsTalkShots decreased perceived risk of infant pertussis. Education strategies based on correcting vaccine misinformation or exposure to fear appeals also have the potential to backfire among those with strong preexisting levels of vaccine hesitancy, [73–75] and thus must be approached with caution and care. However, this decrease in perceived risk of infant pertussis was driven by a reduction in perceived susceptibility among women who received Tdap during pregnancy (Additional file 1: Appendix 5). Perceived severity increased after MomsTalkShots (Additional file 1: Appendix 1). So, women who received Tdap after learning how Tdap protects their infant from pertussis via MomsTalkShots correctly perceived a reduction in susceptibility of their infant to pertussis and correctly identified the increased severity of pertussis for infants.

### Limitations

Our study design included randomization at both the practice/provider and the patient levels. We hypothesized that both interventions might impact KAB constructs, and there might be an improved impact when combined. However, only the patient level intervention (MomsTalkShots) appeared to have an impact; the number of construct associations found with the practice/provider intervention was less than expected by chance alone and the interaction between practice/provider and patient interventions was non-significant. So we focused our per protocol analysis on constructs in the main analysis to reduce redundancy and the chances of type 1 error. There were many insignificant associations that we did not explicitly comment on in this manuscript; they may indicate MomsTalkShots did not impact certain vaccine perceptions, especially among those not intending to vaccinate at baseline. Although we analysed each KAB construct both as a continuous score and a dichotomous indicator of an above-average score, we focused exclusively on the dichotomous analysis in the Results and corresponding tables, despite continuous measures typically providing greater power and precision than dichotomous measures derived from them. However, we found the dichotomous analyses to be much more interpretable and thus more useful for the main text. We have presented both analyses fully in the Appendices for transparency; aside from small differences in significance due to loss of power, the results of the dichotomous analysis (Additional file 1: Appendices 1–2) were highly consistent with the results of the continuous analysis (Additional file 1: Appendices 3–4), justifying our approach. MomsTalkShots’ decrease in perceived risk of infant pertussis being driven by women who received Tdap during pregnancy illustrates the potential effect modification of vaccination, which was not included in our analytic approach beyond further exploration of an unexpected result. Small numbers of women intending their children to receive no recommended vaccines did not provide enough power to justify stratifying by this group alone, so it was combined with those who intended their children to receive some (but not all) recommended vaccines. Although this made our stratification less precise, it increased our power and simplified our analysis. Even with this combination, power was still limited, leading to many potential associations that were close to but not quite statistically significant at the prespecified *p* < 0.05 cutoff. This analysis focused on pregnant women and mothers and thus did not account for others who may be heavily involved in vaccine decisions for children, though data on partners, family and close friends of these pregnant women are published elsewhere [55, 56]. Our sample was comprised mostly of highly educated white women, despite efforts to recruit from a geographically and socio-demographically diverse set of prenatal care practices. Finally, loss to follow-up with differential rates by baseline intention to vaccinate may have biased our data, especially from the final follow-up at 1 year after birth, as those who intended to vaccinate were more likely both to follow-up and to interact positively with MomsTalkShots.

Evaluation of MomsTalkShots through a RCT has high internal validity and provides compelling efficacy data. Our study included recruitment by study coordinators in obstetric practices using financial incentives, whereas in a real-world setting, strategies would be needed to encourage pregnant women and mothers to use MomsTalkShots without these incentives. While scale-up is not a challenge from a technical perspective, dissemination and support from healthcare providers, public health authorities, and other partners would be critical. Other similar interventions do not offer this potential combination of effectiveness and scalability. In-person training of providers to improve their vaccine communication with patients has been shown to be effective, but scale-up would be cost- and time-intensive [52–54]. Several other educational vaccine apps and websites have been developed [70, 71, 76–80]. These include: *ImmunizeCA*, a smartphone app which helps Canadians manage their family’s immunizations by generating customized immunization schedules and reminder alerts for each family member [77]; *ReadyVax*, a smartphone app providing access to evidence-based vaccine information for providers and patients [79]; and *HPV Vaccine: Same Way, Same Day*, a smartphone app which teaches evidence-based vaccine recommendation practices including motivational interviewing skills using simulated role-play scenarios [80]. However, only one – a web-based social media intervention during pregnancy – demonstrated a significant positive effect on vaccine uptake, and its scalability is a challenge due to its reliance on public interactions with vaccine experts, whose time is limited and expensive [70, 71]. MomsTalkShots is the only app or website that tailors information on vaccine attitudes, concerns and demographics. Further research is needed to identify characteristics beyond tailoring that make such apps and websites effective versus ineffective among various populations and settings.

The need for effective interventions to improve vaccine confidence and uptake has only increased since the conclusion of this study. The Coronavirus disease 2019 (COVID-19) pandemic has caused widespread morbidity and mortality, while disjointed government response has led to confusion, the unfortunate politicization of vaccination, and vaccine hesitancy coming to the forefront of public consciousness [81, 82]. COVID-19 vaccines were at first particularly perplexing for pregnant women, given pregnant women’s exclusion from clinical trials but increased risk for severe illness from COVID-19 [83]. As of June 2022, 71% of US pregnant women were fully vaccinated against COVID-19, compared to 77% of adults overall [36]. However, most (95%) vaccinated pregnant women had been fully vaccinated before becoming pregnant. Promising early data on efficacy [84–86] and safety [87–89] of mRNA COVID-19 vaccines in pregnancy were eventually published, and the American College of Obstetricians and Gynecologists (ACOG) strongly supports vaccination against COVID-19 during pregnancy [90].

Adaptation of MomsTalkShots for new vaccines and populations beyond English-speaking pregnant women in the US has the potential to improve vaccine knowledge and perceptions more broadly. We are currently updating and expanding MomsTalkShots to become “LetsTalkShots”, which will cover vaccines across the lifespan, including routine adolescent and adult vaccines such as HPV, influenza, and shingles. Crucially, MomsTalkShots has also been adapted to improve COVID-19 vaccine knowledge and perceptions by providing easily accessible, individually-tailored messages to assuage common concerns about COVID-19 vaccines (e.g., new technology, rushed timeline for development, long-term safety, fertility, safety in pregnancy) and appeal to populations with lower acceptance (e.g., pregnant women, ethnic minorities, younger age, less education, conservative political ideology) [91–93]. This new iteration of MomsTalkShots, called “LetsTalkCovidVaccines”, is free and accessible for all at http://letstalkcovidvaccines.com/. The content, design, and distribution of LetsTalkShots and LetsTalkCovidVaccines will be regularly assessed and upgraded, to reflect updates in science, incorporate new topics of concern, improve the user experience, and expand access, reach, and impact.

## Conclusions

MomsTalkShots improved vaccine knowledge and perceptions among pregnant women and mothers. Among women initially intending not to vaccinate, MomsTalkShots increased perceived risk of maternal influenza disease and confidence in influenza vaccine efficacy; and among women with uncertain infant vaccine intentions, MomsTalkShots substantially reduced safety concerns. MomsTalkShots offers a scalable tool to address vaccine hesitancy by disseminating easily accessible information tailored to individuals’ demographics and concerns, and is currently being updated to cover vaccines across the lifespan.

## Supplementary Information

**Additional file 1: Appendix 1.** Impact of MomsTalkShots on Vaccine KABs of Women One Month After Their Infant's Birth individual survey items, organized by constructs, stratified by baseline vaccine intentions^a^, dichotomous analysis^b^. **Appendix 2.** Impact of MomsTalkShots on Vaccine KABs of Women One Year After Their Infant's Birth individual survey items, organized by constructs, stratified by baseline vaccine intentions^a^, dichotomous analysis^b^. **Appendix 3.** Impact of MomsTalkShots on Vaccine KABs of Women One Month After Their Infant's Birth individual survey items, organized by constructs, stratified by baseline vaccine intentions^a^, continuous analysis^b^. **Appendix 4.** Impact of MomsTalkShots on Vaccine KABs of Women One Year After Their Infant's Birth individual survey items, organized by constructs, stratified by baseline vaccine intentions^a^, continuous analysis^b^. **Appendix 5.** Impact of MomsTalkShots on Women's Perceived Risk of Infant Pertussis One Month After Their Infant's Birth individual survey items, stratified by baseline vaccine intentions^a^ and Tdap vaccination, dichotomous analysis^b^. **Appendix 6.** Impact of MomsTalkShots on Women's Perceived Risk of Infant Pertussis One Year After Their Infant's Birth individual survey items, stratified by baseline vaccine intentions^a^ and Tdap vaccination, dichotomous analysis^b^. **Appendix 7.** Survey Initiation and Completion stratified by study arm. **Appendix 8.** Follow-Up Survey Completion stratified by maternal and infant vaccine intentions at baseline. **Appendix 9.** Response Rates for Vaccine KAB Constructs at Each Study Timepoint. **Appendix 10.** Maternal and Infant Vaccine Intentions at Baseline stratified by 4 study arms.

## Data Availability

The datasets generated and analysed during our study are not publicly available due to confidentiality issues, but may be requested from the corresponding author.

## Abbreviations

US: United States
(Tdap) vaccination: Tetanus, diphtheria, and acellular pertussis
COVID-19: Coronavirus disease 2019
KABs: Knowledge, attitudes and beliefs
app: Application
CDC: Centers for Disease Control and Prevention
(AFIX) model: Assessment, Feedback, Incentives and Exchange
CME: Continuing Medical Education
IRB: Institutional Review Board
