# Supplementary material for: MomsTalkShots, tailored educational app, improves vaccine attitudes: a randomized controlled trial
Source: BMC Public Health. 2022 Nov 21;22:2134. doi: 10.1186/s12889-022-14498-7 (PMC9676851; doi:10.1186/s12889-022-14498-7)
Supplement: Supplementary file 1 — Additional file 1: Appendix 1. Impact of MomsTalkShots on Vaccine KABs of Women One Month After Their Infant's Birth individual survey items, organized by constructs, stratified by baseline vaccine intentionsa, dichotomous analysisb. Appendix 2. Impact of MomsTalkShots on Vaccine KABs of Women One Year After Their Infant's Birth individual survey items, organized by constructs, stratified by baseline vaccine intentionsa, dichotomous analysisb. Appendix 3. Impact of MomsTalkShots on Vaccine KABs of Women One Month After Their Infant's Birth individual survey items, organized by constructs, stratified by baseline vaccine intentionsa, continuous analysisb. Appendix 4. Impact of MomsTalkShots on Vaccine KABs of Women One Year After Their Infant's Birth individual survey items, organized by constructs, stratified by baseline vaccine intentionsa, continuous analysisb. Appendix 5. Impact of MomsTalkShots on Women's Perceived Risk of Infant Pertussis One Month After Their Infant's Birth individual survey items, stratified by baseline vaccine intentionsa and Tdap vaccination, dichotomous analysisb. Appendix 6. Impact of MomsTalkShots on Women's Perceived Risk of Infant Pertussis One Year After Their Infant's Birth individual survey items, stratified by baseline vaccine intentionsa and Tdap vaccination, dichotomous analysisb. Appendix 7. Survey Initiation and Completion stratified by study arm. Appendix 8. Follow-Up Survey Completion stratified by maternal and infant vaccine intentions at baseline. Appendix 9. Response Rates for Vaccine KAB Constructs at Each Study Timepoint. Appendix 10. Maternal and Infant Vaccine Intentions at Baseline stratified by 4 study arms. [file 12889_2022_14498_MOESM1_ESM.docx]

**Appendices**

**Appendix 1. Impact of MomsTalkShots on Vaccine KABs of Women One Month After Their Infant's Birth**

individual survey items, organized by constructs, stratified by baseline vaccine intentions^a^, dichotomous analysis^b^

|  | % agreeing with statements or with above average construct scores^c^ among those **not** receiving MomsTalkShots | | | | % agreeing with statements or with above average construct scores^c^ among those receiving MomsTalkShots | | | | Effect of MomsTalkShots on % agreeing with statements or with above average construct scores^c^,  OR (95% CI)^b^ | | | |
| --- | --- | --- | --- | --- | --- | --- | --- | --- | --- | --- | --- | --- |
| *Intentions to Vaccinate^a^* | *All* | *Yes* | *No* | *Unsure* | *All* | *Yes* | *No* | *Unsure* | *All* | *Yes* | *No* | *Unsure* |
| ***KAB Constructs* and their Constituent Statements^c^** |  |  |  |  |  |  |  |  |  |  |  |  |
| *Confidence in vaccine safety (for the infant)* |  |  |  |  |  |  |  |  |  |  |  |  |
| I am confident that vaccines are safe for my baby^4^ | 88 | 95 | 53 | 57 | 88 | 96 | 36 | 58 | 0.96 (0.66-1.39) | 1.37 (0.77-2.45) | 0.49 (0.23-1.04) | 1.04 (0.46-2.36) |
| *Specific safety concerns (for infant vaccines)^4e^* | 56 | 49 | 90 | 89 | 53 | 47 | 87 | 81 | 0.89 (0.70-1.12) | 0.94 (0.73-1.20) | 0.62 (0.19-2.05) | 0.44 (0.14-1.40) |
| It is better for babies to get fewer vaccines at the same time^4e^ | 10 | 4 | 37 | 31 | 10 | 3 | 37 | 37 | 1.10 (0.73-1.65) | 0.64 (0.33-1.22) | 2.15 (0.99-4.69) | 1.30 (0.57-2.99) |
| Babies get more vaccines in their first two years of life than are good for them^4e^ | 8 | 3 | 35 | 30 | 9 | 3 | 49 | 33 | 1.06 (0.69-1.61) | 0.79 (0.39-1.59) | 1.76 (0.79-3.94) | 1.05 (0.45-2.46) |
| Vaccines often cause serious side effects in babies^4e^ | 8 | 3 | 32 | 33 | 8 | 2 | 49 | 27 | 0.88 (0.57-1.38) | 0.71 (0.34-1.50) | 2.15 (0.92-5.07) | 0.58 (0.24-1.43) |
| The ingredients in vaccines are not safe for my baby^4e^ | 9 | 3 | 41 | 31 | 9 | 3 | 58 | 23 | 0.94 (0.61-1.46) | 0.86 (0.42-1.74) | 1.97 (0.86-4.48) | 0.51 (0.19-1.33) |
| *Perceived risk (maternal influenza)^1^* | 55 | 64 | 32 | 42 | 61 | 71 | 38 | 44 | **1.61 (1.23-2.09)** | **1.57 (1.12-2.20)** | **2.07 (1.15-3.71)** | 1.53 (0.70-3.34) |
| The flu is dangerous for pregnant women^1^ | 86 | 89 | 78 | 81 | 87 | 90 | 80 | 84 | 1.15 (0.79-1.67) | 1.07 (0.65-1.77) | 1.29 (0.64-2.63) | 1.41 (0.49-4.07) |
| The flu is more dangerous for pregnant women than for women who are not pregnant^1^ | 83 | 88 | 74 | 70 | 85 | 90 | 77 | 75 | **1.66 (1.13- 2.44)** | 1.54 (0.91-2.60) | 1.55 (0.73- 3.29) | **2.90 (1.01-8.33)** |
| *Confidence in vaccine efficacy (maternal influenza vaccine)^1^* | 63 | 77 | 28 | 39 | 73 | 86 | 44 | 52 | **1.97 (1.47-2.65)** | **2.09 (1.36-3.22)** | **2.62 (1.46-4.69)** | 1.96 (0.90-4.26) |
| Getting the flu vaccine will reduce (reduced/would have reduced) my risk of getting the flu during my pregnancy^1^ | 74 | 89 | 37 | 48 | 79 | 93 | 52 | 53 | **1.60 (1.12-2.28)** | 1.46 (0.83-2.57) | **2.46 (1.35-4.47)** | 1.34 (0.59-3.04) |
| Getting the flu vaccine while I am pregnant will reduce (reduced/would have reduced) my baby’s risk of getting the flu^1^ | 64 | 77 | 28 | 51 | 75 | 87 | 48 | 60 | **2.28 (1.68-3.09)** | **2.50 (1.60-3.89)** | **3.28 (1.81-5.95)** | 1.70 (0.77-3.77) |
| *Perceived risk (maternal whooping cough)* |  |  |  |  |  |  |  |  |  |  |  |  |
| Whooping cough is dangerous for pregnant women^2^ | 83 | 86 | 76 | 78 | 85 | 87 | 80 | 77 | 1.32 (0.91-1.91) | 1.17 (0.72-1.90) | 1.40 (0.67-2.93) | 2.03 (0.69-6.00) |
| *Perceived risk (infant whooping cough)^4^* | 42 | 47 | 22 | 19 | 39 | 42 | 19 | 31 | **0.75 (0.57-1.00)^f^** | **0.70 (0.51-0.96)** | 0.77 (0.27-2.24) | 2.18 (0.68-6.96) |
| I worry that my baby could get whooping cough^4^ | 51 | 55 | 30 | 24 | 47 | 49 | 33 | 37 | 0.78 (0.59-1.03) | **0.72 (0.53-0.98)** | 0.78 (0.31-2.01) | 1.98 (0.67-5.85) |
| Whooping cough is dangerous for babies^4^ | 96 | 97 | 90 | 98 | 98 | 98 | 98 | 85 | **2.69 (1.14-6.37)** | 1.59 (0.61-4.15) | 3.83 (0.45-32.72) | N/A^d^ |
| Whooping cough is more dangerous for babies than older children or adults^4^ | 93 | 95 | 86 | 81 | 94 | 94 | 91 | 90 | 1.15 (0.68-1.93) | 0.86 (0.47-1.57) | 3.19 (0.63-16.22) | 2.26 (0.44-11.51) |
| *Confidence in vaccine efficacy (whooping cough vaccine)^2^* | 56 | 68 | 24 | 47 | 61 | 72 | 37 | 41 | 1.01 (0.77-1.33) | 0.96 (0.69-1.34) | 1.42 (0.71-2.86) | 0.53 (0.18-1.52 |
| Whooping cough vaccine will reduce (reduced/would have reduced) my chances of getting whooping cough^2^ | 80 | 90 | 52 | 63 | 84 | 91 | 67 | 75 | 1.61 (0.82-1.65) | 0.88 (0.54-1.45) | 1.42 (0.73-2.77) | 1.51 (0.68-3.34) |
| Whooping cough vaccine will reduce (reduced/would have reduced) the chance of me giving whooping cough to my baby^2^ | 84 | 92 | 60 | 76 | 87 | 95 | 67 | 80 | 0.97 (0.66-1.42) | 1.06 (0.59-1.92) | 1.01 (0.53-1.92) | 1.25 (0.48-3.28) |
| Getting the whooping cough vaccine while I am pregnant will reduce (reduced/would have reduced) my baby’s risk of getting whooping cough^2^ | 81 | 90 | 56 | 67 | 86 | 93 | 66 | 78 | 1.08 (0.76-1.55) | 0.97 (0.58-1.61) | 1.16 (0.62-2.17) | 1.73 (0.71-4.21) |
| Getting the whooping cough vaccine for my baby will reduce my baby’s chances of getting whooping cough^4^ | 86 | 91 | 61 | 61 | 88 | 94 | 51 | 67 | 1.01 (0.68-1.50) | 1.14 (0.67-1.94) | 0.77 (0.33-1.80) | 1.01 (0.33-3.05) |
| *Belief in superiority of natural immunity^e^* |  |  |  |  |  |  |  |  |  |  |  |  |
| I believe it is better for my baby to develop their own immunity by getting sick rather than by getting a vaccine^4e^ | 22 | 16 | 47 | 46 | 24 | 18 | 55 | 47 | 1.03 (0.75-1.43) | 1.07 (0.73-1.57) | 0.72 (0.30-1.74) | 0.94 (0.33-2.64) |
| *Pro-vaccine social norms^4^* | 44 | 49 | 19 | 15 | 46 | 51 | 15 | 33 | 1.08 (0.86-1.35) | 1.05 (0.83-1.34) | 0.55 (0.20-1.47) | 2.57 (0.97-6.85) |
| The majority of my friends and family would get all of the vaccines recommended for their babies after the birth^4^ | 86 | 91 | 65 | 59 | 88 | 92 | 62 | 65 | 1.15 (0.83-1.58) | 1.21 (0.80-1.83) | 0.81 (0.39-1.67) | 1.23 (0.51-2.96) |
| The majority of my friends and family would encourage me to get all of the vaccines recommended for my baby^4^ | 85 | 90 | 61 | 57 | 87 | 92 | 56 | 65 | 1.17 (0.85-1.61) | 1.28 (0.85-1.93) | 0.77 (0.38-1.58) | 1.30 (0.53-3.17 |
| *Self-efficacy* |  |  |  |  |  |  |  |  |  |  |  |  |
| It is in my control whether or not my baby gets his/her vaccines^4^ | 96 | 98 | 95 | 83 | 97 | 98 | 91 | 92 | 1.75 (0.78-3.89) | 1.80 (0.59-5.51) | 0.58 (0.12-2.89) | 5.67 (0.67-48.36) |
| *Perceived vaccine knowledge^4^* | 48 | 53 | 24 | 28 | 55 | 59 | 38 | 33 | **1.39 (1.13-1.72)** | **1.36 (1.08-1.70)** | 2.04 (0.97-4.28) | 1.00 (0.41-2.45) |
| I have most of the important information I need to make a decision about vaccines for my baby^4^ | 93 | 95 | 80 | 83 | 94 | 97 | 82 | 76 | 1.37 (0.89-2.11) | **1.89 (1.02-3.49)** | 1.36 (0.52-3.55) | 0.62 (0.23-1.67) |
| I know enough about the safety of the whooping cough vaccine to make a decision about getting the vaccine for my baby^4^ | 88 | 91 | 73 | 74 | 91 | 94 | 79 | 77 | **1.49 (1.05-2.09)** | **1.63 (1.06-2.51)** | 1.47 (0.62-3.50) | 1.03 (0.41-2.55) |
| *Trust in vaccine information (from obstetricians and pediatricians)^4^* | 53 | 60 | 23 | 20 | 59 | 27 | 26 | 27 | 1.22 (0.97-1.54) | 1.22 (0.95-1.56) | 1.43 (0.63-3.27) | 1.12 (0.43-2.93) |
| I trust the information provided by my obstetrician or midwife about vaccines during pregnancy^3^ | 92 | 96 | 74 | 89 | 93 | 97 | 73 | 85 | 1.25 (0.81- 1.92) | 1.42 (0.74-2.73) | 1.02 (0.47-2.21) | 0.98 (0.37-2.23) |
| I trust the information provided by my obstetrician or midwife about vaccines for babies^4^ | 90 | 95 | 65 | 65 | 93 | 98 | 62 | 67 | **1.62 (1.09- 2.43)** | **2.70 (1.33-5.49)** | 1.08 (0.51- 2.30) | 1.18 (0.52-2.71) |
| I trust the information provided by my baby's doctor about vaccines during pregnancy^3^ | 92 | 96 | 72 | 85 | 93 | 97 | 73 | 83 | 0.89 (0.56-1.42) | 0.76 (0.37-1.57) | 1.13 (0.48-2.65) | 0.89 (0.32-2.54) |
| I trust the information provided by my baby's doctor about vaccines for babies^4^ | 93 | 98 | 65 | 70 | 93 | 99 | 66 | 63 | 1.10 (0.68-1.80) | 1.71 (0.67-4.38) | 1.49 (0.60-3.73) | 0.49 (0.18-1.37) |
| *Trust in vaccine information (from naturopaths and chiropractors)^4^* | 56 | 57 | 60 | 51 | 60 | 61 | 59 | 51 | 0.83 (0.57-1.19) | 0.76 (0.50-1.16) | 1.04 (0.33-3.30) | 0.83 (0.26-2.61) |
| I trust the information provided by naturopathic and/or chiropractic doctors about vaccines during pregnancy^3^ | 63 | 62 | 70 | 57 | 68 | 70 | 72 | 54 | 1.02 (0.69-1.53) | 1.20 (0.74-1.97) | 0.73 (0.25-2.13) | 0.67 (0.26-1.72) |
| I trust the information provided by naturopathic and/or chiropractic doctors about vaccines for babies^4^ | 63 | 63 | 66 | 61 | 66 | 67 | 65 | 58 | 0.78 (0.50-1.22) | 0.81 (0.48-1.36) | 0.67 (0.19 -2.40) | 0.60 (0.15-2.38) |
| *Trust in vaccine information (from federal agencies and academic institutions)^4^* | 48 | 55 | 18 | 13 | 49 | 55 | 14 | 25 | 1.06 (0.84-1.34) | 1.02 (0.79-1.31) | 0.77 (0.29-2.06) | 1.88 (0.65-5.47) |
| I trust the information provided by federal agencies such as the Centers for Disease Control and Prevention (CDC) about vaccines during pregnancy^3^ | 84 | 90 | 55 | 73 | 84 | 91 | 48 | 72 | 1.00 (0.73-1.37) | 1.11 (0.73-1.69) | 0.70 (0.36-1.37) | 1.06 (0.51-2.21) |
| I trust the information provided by federal agencies such as the Centers for Disease Control and Prevention (CDC) about vaccines for babies^4^ | 83 | 91 | 47 | 58 | 84 | 92 | 33 | 68 | 1.05 (0.77-1.43) | 1.16 (0.77-1.76) | 0.52 (0.23-1.18) | 1.51 (0.53-4.26) |
| I trust the information provided by scientists and doctors at universities and academic institutions about vaccines during pregnancy^3^ | 86 | 92 | 58 | 78 | 85 | 91 | 50 | 78 | 0.96 (0.70-1.31) | 1.03 (0.67-1.58) | 0.62 (0.31-1.24) | 1.02 (0.45-2.30) |
| I trust the information provided by scientists and doctors at universities and academic institutions about vaccines for babies^4^ | 86 | 92 | 58 | 67 | 85 | 90 | 52 | 62 | 0.90 (0.66-1.24) | 0.91 (0.61-1.36) | 0.84 (0.41-1.69) | 0.65 (0.26-1.61) |

^a^Constructs are italicized, with constituent statements listed below. For constructs and statements specific to maternal influenza disease or vaccine, "intend to vaccinate", "intend not to vaccinate", and "uncertain intentions" refer to maternal influenza vaccine^1^; for constructs and statements specific to maternal pertussis disease or vaccine, "intend to vaccinate", "intend not to vaccinate", and "uncertain intentions" refer to maternal Tdap vaccine^2^; for constructs and statements relevant to both maternal influenza and pertussis diseases/vaccines, "intend to vaccinate", "intend not to vaccinate", and "uncertain intentions" refer to at least one of maternal influenza and Tdap vaccines^3^; for constructs and statements specific to infant diseases or vaccines, "intend to vaccinate", "intend not to vaccinate", and "uncertain intentions" refer to intending to receive all recommended infant vaccines versus intending to receive some or no recommended infant vaccines^4^; for constructs and statements relevant to both maternal and infant vaccines, "intend to vaccinate", "intend not to vaccinate", and "uncertain intentions" refer to intending to receive all recommended infant vaccines versus intending to receive some or no recommended infant vaccines^4^; and for constructs with constituent statements belonging to different of the above categories, "intend to vaccinate", "intend not to vaccinate", and "uncertain intentions" refer to the most common category, and in the case of a tie refer to intending to receive all recommended infant vaccines versus intending to receive some or no recommended infant vaccines^4^.

^b^OR = Odds Ratio (from logistic regression); 95%CI = 95% Confidence Interval; bolded if statistically significant.

^c^KAB = knowledge, attitudes, and beliefs. Likert scale responses were encoded as follows: 1 - strongly disagree, 2 - disagree, 3 - don’t know (only included for items assessing knowledge or trust), 4 - agree, 5 - strongly agree. Summary scores were created for all KAB constructs with multiple constituent survey statements at each timepoint. Dichotomous variables assessing construct summary scores coded scores above the average as 1 and scores below the average as 0.

^d^N/A = not applicable (n=1 in a cell of 2-by-2 table; p>0.99)

^e^Negatively phrased construct or statement, for which a negative association would indicate a positive effect on vaccine KAB (other constructs and statements were created from positively phrased survey statements, for which a positive association would indicate a positive effect on vaccine KAB)

^f^This appearance of an overlap with 1 in the 95%CI is due to rounding

**Appendix 2. Impact of MomsTalkShots on Vaccine KABs of Women One Year After Their Infant's Birth**

individual survey items, organized by constructs, stratified by baseline vaccine intentions^a^, dichotomous analysis^b^

|  | % agreeing with statements or with above average construct scores^c^ among those **not** receiving MomsTalkShots | | | | % agreeing with statements or with above average construct scores^c^ among those receiving MomsTalkShots | | | | Effect of MomsTalkShots on % agreeing with statements or with above average construct scores^c^,  OR (95% CI)^b^ | | | |
| --- | --- | --- | --- | --- | --- | --- | --- | --- | --- | --- | --- | --- |
| *Intentions to Vaccinate^a^* | *All* | *Yes* | *No* | *Unsure* | *All* | *Yes* | *No* | *Unsure* | *All* | *Yes* | *No* | *Unsure* |
| ***KAB Constructs* and their Constituent Statements^c^** |  |  |  |  |  |  |  |  |  |  |  |  |
| *Confidence in vaccine safety (for the infant)* |  |  |  |  |  |  |  |  |  |  |  |  |
| I am confident that vaccines are safe for my baby^4^ | 90 | 95 | 60 | 70 | 92 | 97 | 52 | 68 | 1.29 (0.78-2.15) | 1.81 (0.86-3.80) | 0.78 (0.31-1.97) | 1.39 (0.37-5.22) |
| *Specific safety concerns (for infant vaccines)^4e^* | 47 | 39 | 83 | 91 | 44 | 38 | 93 | 71 | 0.88 (0.67-1.15) | 0.93 (0.70-1.25) | 2.78 (0.67-11.46) | **0.24 (0.06-0.98)** |
| It is better for babies to get fewer vaccines at the same time^4e^ | 9 | 5 | 34 | 21 | 7 | 2 | 45 | 29 | 0.77 (0.45-1.31) | 0.48 (0.22-1.05) | 1.53 (0.60-3.91) | 1.37 (0.39-4.84) |
| Babies get more vaccines in their first two years of life than are good for them^4e^ | 7 | 3 | 34 | 21 | 6 | 1 | 38 | 29 | 0.73 (0.42-1.27) | 0.46 (0.18-1.20) | 0.95 (0.38-2.38) | 1.02 (0.26-3.97) |
| Vaccines often cause serious side effects in babies^4e^ | 7 | 3 | 31 | 15 | 5 | 1 | 36 | 21 | 0.55 (0.29-1.04) | **0.31 (0.10-0.98)** | 0.89 (0.32-2.45) | 0.86 (0.20-3.66) |
| The ingredients in vaccines are not safe for my baby^4e^ | 8 | 3 | 34 | 30 | 7 | 2 | 45 | 29 | 0.71 (0.39-1.29) | 0.58 (0.23-1.46) | 0.98 (0.31-3.17) | 0.65 (0.18-2.33) |
| *Perceived risk (infant whooping cough)^4^* | 46 | 49 | 26 | 24 | 39 | 40 | 19 | 44 | 0.89 (0.66-1.21) | 0.89 (0.64-1.22) | 0.53 (0.13-2.27) | 1.05 (0.27-4.08) |
| I worry that my baby could get whooping cough^4^ | 55 | 57 | 38 | 42 | 46 | 47 | 31 | 50 | 0.79 (0.58-1.08) | 0.80 (0.58-1.11) | 0.85 (0.25-2.91) | 0.50 (0.13-1.92) |
| Whooping cough is dangerous for babies^4^ | 96 | 98 | 88 | 88 | 97 | 97 | 95 | 97 | 1.37 (0.60-3.13) | 0.78 (0.30-2.04) | 3.04 (0.32-28.72) | N/A^d^ |
| Whooping cough is more dangerous for babies than older children or adults^4^ | 93 | 94 | 79 | 88 | 95 | 96 | 83 | 97 | **1.98 (1.02-3.87)** | 1.96 (0.86-4.47) | 1.18 (0.31-4.40) | N/A^d^ |
| *Confidence in vaccine efficacy (whooping cough vaccine)* |  |  |  |  |  |  |  |  |  |  |  |  |
| Getting the whooping cough vaccine for my baby will reduce (reduced/would have reduced) my baby’s chances of getting whooping cough^4^ | 91 | 96 | 67 | 67 | 91 | 95 | 60 | 76 | 0.87 (0.51-1.49) | 0.75 (0.35-1.58) | 1.15 (0.34-3.83) | 0.63 (0.14-2.82) |
| *Belief in superiority of natural immunity^e^* |  |  |  |  |  |  |  |  |  |  |  |  |
| I believe it is better for my baby to develop their own immunity by getting sick rather than by getting a vaccine^4e^ | 17 | 14 | 36 | 30 | 20 | 17 | 43 | 35 | 1.21 (0.82-1.80) | 1.25 (0.80-1.97) | 1.18 (0.41-3.43) | 0.97 (0.21-4.40) |
| *Pro-vaccine social norms^4^* | 49 | 54 | 22 | 21 | 55 | 59 | 21 | 38 | 1.23 (0.95-1.58) | 1.22 (0.93-1.61) | 0.67 (0.23-1.94) | 2.13 (0.70-6.53) |
| The majority of my friends and family would get all of the vaccines recommended for their babies after the birth^4^ | 89 | 92 | 66 | 82 | 91 | 95 | 67 | 74 | 1.33 (0.87-2.02) | 1.50 (0.88-2.56) | 1.05 (0.39-2.81) | 0.75 (0.19-2.99) |
| The majority of my friends and family would encourage me to get all of the vaccines recommended for my baby^4^ | 84 | 89 | 60 | 67 | 89 | 93 | 57 | 68 | **1.56 (1.07-2.27)** | **1.92 (1.20-1.38)** | 0.86 (0.33-2.23) | 1.29 (0.38-4.38) |
| *Self-efficacy* |  |  |  |  |  |  |  |  |  |  |  |  |
| It is in my control whether or not my baby gets his/her vaccines^4^ | 94 | 96 | 90 | 73 | 98 | 98 | 95 | 97 | 2.00 (0.89-4.49) | 1.45 (0.54-3.88) | 1.19 (0.20-7.13) | N/A^d^ |
| *Perceived vaccine knowledge^4^* | 50 | 55 | 29 | 12 | 62 | 65 | 40 | 47 | **1.74 (1.36-2.24)** | **1.60 (1.22-2.10)** | 1.71 (0.73-4.01) | **6.89 (1.52-31.25)** |
| I have most of the important information I need to make a decision about vaccines for my baby^4^ | 94 | 95 | 90 | 76 | 97 | 98 | 95 | 91 | **2.63 (1.39-4.96)** | **2.36 (1.10-5.07)** | 2.36 (0.44-12.55) | 3.27 (0.65-16.50) |
| I know enough about the safety of the whooping cough vaccine to make a decision about getting the vaccine for my baby^4^ | 90 | 93 | 83 | 73 | 94 | 95 | 90 | 88 | **2.02 (1.25-3.26)** | **1.95 (1.11-3.44)** | 2.04 (0.59-7.10) | 3.32 (0.62-17.81) |
| *Trust in vaccine information (from obstetricians and pediatricians)^4^* | 55 | 63 | 16 | 15 | 64 | 70 | 14 | 41 | **1.53 (1.17-2.00)** | **1.47 (1.09-1.97)** | 1.07 (0.32-3.55) | 3.41 (1.03-11.29) |
| I trust the information provided by my obstetrician or midwife about vaccines during pregnancy^3^ | 93 | 96 | 71 | 91 | 95 | 98 | 77 | 89 | **1.89 (1.07-3.37)** | 2.04 (0.84-4.92) | 1.92 (0.69-5.36) | 1.17 (0.30-4.53) |
| I trust the information provided by my obstetrician or midwife about vaccines for babies^4^ | 92 | 96 | 62 | 82 | 94 | 97 | 62 | 88 | 1.61 (0.97-2.68) | 0.98 (0.46-2.06) | 1.43 (0.52-3.89) | 2.37 (0.52-10.82) |
| I trust the information provided by my baby's doctor about vaccines during pregnancy^3^ | 92 | 95 | 75 | 88 | 94 | 98 | 75 | 86 | 1.42 (0.79-2.56) | 1.98 (0.83-4.74) | 1.61 (0.52-4.99) | 0.52 (0.12-2.23) |
| I trust the information provided by my baby's doctor about vaccines for babies^4^ | 93 | 97 | 69 | 76 | 95 | 99 | 64 | 85 | 1.48 (0.78-2.83) | 1.86 (0.60-5.82) | 0.99 (0.36-2.76) | 1.65 (0.39-6.90) |
| *Trust in vaccine information (from naturopaths and chiropractors)^4^* | 53 | 50 | 62 | 65 | 56 | 56 | 57 | 59 | 1.04 (0.68-1.60) | 1.06 (0.66-1.72) | 0.89 (0.22-3.62) | 0.90 (0.18-4.63) |
| I trust the information provided by naturopathic and/or chiropractic doctors about vaccines during pregnancy^3^ | 60 | 57 | 66 | 71 | 66 | 64 | 69 | 72 | 1.14 (0.71-1.84) | 1.06 (0.59-1.91) | 1.71 (0.52-5.57) | 0.78 (0.21-2.84) |
| I trust the information provided by naturopathic and/or chiropractic doctors about vaccines for babies^4^ | 59 | 57 | 67 | 64 | 64 | 63 | 63 | 67 | 1.00 (0.62-1.63) | 0.90 (0.52-1.56) | 1.53 (0.31-7.54) | 1.34 (0.25-7.10) |
| *Trust in vaccine information (from federal agencies and academic institutions)^4^* | 51 | 58 | 14 | 12 | 54 | 59 | 12 | 32 | 1.30 (0.99-1.71) | 1.21 (0.90-1.62) | 0.93 (0.27-3.24) | 3.03 (0.82-11.12) |
| I trust the information provided by federal agencies such as the Centers for Disease Control and Prevention (CDC) about vaccines during pregnancy^3^ | 87 | 92 | 62 | 86 | 89 | 94 | 60 | 78 | 1.23 (0.81-1.86) | 1.74 (0.97-3.11) | 0.67 (0.30-1.53) | 0.78 (0.27-2.26) |
| I trust the information provided by federal agencies such as the Centers for Disease Control and Prevention (CDC) about vaccines for babies^4^ | 86 | 92 | 55 | 58 | 89 | 94 | 48 | 68 | 1.41 (0.94-2.13) | 1.60 (0.93-2.74) | 0.76 (0.30-1.93) | 1.51 (0.53-4/26 |
| I trust the information provided by scientists and doctors at universities and academic institutions about vaccines during pregnancy^3^ | 88 | 92 | 63 | 84 | 89 | 92 | 74 | 84 | 1.30 (0.87-1.93) | 1.07 (0.63-1.82) | 1.89 (0.83-4.33) | 1.10 (0.39-3.05) |
| I trust the information provided by scientists and doctors at universities and academic institutions about vaccines for babies^4^ | 88 | 92 | 64 | 73 | 90 | 93 | 67 | 68 | 1.33 (0.88-2.00) | 1.28 (0.76-2.15) | 2.35 (0.81-6.81) | 0.68 (0.22-2.12) |

^a^Constructs are italicized, with constituent statements listed below. For constructs and statements specific to maternal influenza disease or vaccine, "intend to vaccinate", "intend not to vaccinate", and "uncertain intentions" refer to maternal influenza vaccine^1^; for constructs and statements specific to maternal pertussis disease or vaccine, "intend to vaccinate", "intend not to vaccinate", and "uncertain intentions" refer to maternal Tdap vaccine^2^; for constructs and statements relevant to both maternal influenza and pertussis diseases/vaccines, "intend to vaccinate", "intend not to vaccinate", and "uncertain intentions" refer to at least one of maternal influenza and Tdap vaccines^3^; for constructs and statements specific to infant diseases or vaccines, "intend to vaccinate", "intend not to vaccinate", and "uncertain intentions" refer to intending to receive all recommended infant vaccines versus intending to receive some or no recommended infant vaccines^4^; for constructs and statements relevant to both maternal and infant vaccines, "intend to vaccinate", "intend not to vaccinate", and "uncertain intentions" refer to intending to receive all recommended infant vaccines versus intending to receive some or no recommended infant vaccines^4^; and for constructs with constituent statements belonging to different of the above categories, "intend to vaccinate", "intend not to vaccinate", and "uncertain intentions" refer to the most common category, and in the case of a tie refer to intending to receive all recommended infant vaccines versus intending to receive some or no recommended infant vaccines^4^.

^b^OR = Odds Ratio (from logistic regression); 95%CI = 95% Confidence Interval; bolded if statistically significant.

^c^KAB = knowledge, attitudes, and beliefs. Likert scale responses were encoded as follows: 1 - strongly disagree, 2 - disagree, 3 - don’t know (only included for items assessing knowledge or trust), 4 - agree, 5 - strongly agree. Summary scores were created for all KAB constructs with multiple constituent survey statements at each timepoint. Dichotomous variables assessing construct summary scores coded scores above the average as 1 and scores below the average as 0.

^d^N/A = not applicable (n=1 in a cell of 2-by-2 table; p>0.99)

^e^Negatively phrased construct or statement, for which a negative association would indicate a positive effect on vaccine KAB (other constructs and statements were created from positively phrased survey statements, for which a positive association would indicate a positive effect on vaccine KAB)

**Appendix 3. Impact of MomsTalkShots on Vaccine KABs of Women One Month After Their Infant's Birth**

individual survey items, organized by constructs, stratified by baseline vaccine intentions^a^, continuous analysis^b^

|  | Average scaled scores^c^ among those **not** receiving MomsTalkShots | | | | Average scaled scores^c^ among those receiving MomsTalkShots | | | | Effect of MomsTalkShots on average scaled scores^c^, RC (95% CI)** | | | |
| --- | --- | --- | --- | --- | --- | --- | --- | --- | --- | --- | --- | --- |
| *Intentions to Vaccinate^a^* | *All* | *Yes* | *No* | *Unsure* | *All* | *Yes* | *No* | *Unsure* | *All* | *Yes* | *No* | *Unsure* |
| ***KAB Constructs* and their Constituent Statements^c^** |  |  |  |  |  |  |  |  |  |  |  |  |
| *Confidence in vaccine safety (for the infant)* |  |  |  |  |  |  |  |  |  |  |  |  |
| I am confident that vaccines are safe for my baby^4^ | 83.19 | 87.86 | 59.75 | 61.85 | 83.92 | 89.05 | 50.75 | 65.00 | 0.09 (-1.53-1.70) | 0.51 (-0.96-1.98) | -6.19 (-13.57-1.19) | 4.44 (-5.00-13.88) |
| *Specific safety concerns (for infant vaccines)^4d^* | 35.25 | 31.44 | 54.87 | 52.31 | 34.86 | 30.51 | 63.73 | 50.10 | -0.18 (-1.56-1.21) | -0.48 (-1.76-0.81) | 5.12 (-0.97-11.21) | -3.60 (-11.91-4.71) |
| It is better for babies to get fewer vaccines at the same time^4d^ | 35.59 | 31.93 | 54.68 | 51.11 | 35.16 | 30.63 | 64.18 | 52.31 | 0.10 (-1.48-1.68) | -0.83 (-2.26-0.60) | 6.81 (-0.53-14.16) | 1.48 (-8.13-11.10) |
| Babies get more vaccines in their first two years of life than are good for them^4d^ | 35.05 | 31.28 | 55.19 | 51.11 | 34.89 | 30.60 | 61.79 | 51.92 | 0.11 (-1.43-1.65) | -0.20 (-1.53-1.13) | 3.43 (-4.11-10.97) | -0.36 (-10.21-9.49) |
| Vaccines often cause serious side effects in babies^4d^ | 34.95 | 31.28 | 52.41 | 53.70 | 34.59 | 30.34 | 62.99 | 49.23 | -0.36 (-1.83-1.11) | -0.52 (-1.83-0.80) | **7.11 (0.43-13.78)** | -7.01 (-16.48-2.46) |
| The ingredients in vaccines are not safe for my baby^4d^ | 35.41 | 31.28 | 57.22 | 53.33 | 34.59 | 30.47 | 66.97 | 46.92 | -0.48 (-1.95-1.00) | -0.41 (-1.72-0.91) | 5.69 (-1.42-12.79) | -8.32 (-17.06-0.43) |
| *Perceived risk (maternal influenza)^1^* | 84.42 | 87.02 | 77.32 | 81.00 | 85.67 | 88.63 | 79.49 | 80.00 | **2.57 (0.50-4.64)** | 2.13 (-0.31-4.58) | **4.83 (0.19-9.48)** | 1.66 (-4.73-8.06) |
| The flu is dangerous for pregnant women^1^ | 85.46 | 88.01 | 78.09 | 82.89 | 86.36 | 89.03 | 80.76 | 81.36 | 1.28 (-1.20-3.77) | 0.87 (-2.15-3.89) | 4.51 (-0.86-9.87) | -1.27 (-8.41-5.86) |
| The flu is more dangerous for pregnant women than for women who are not pregnant^1^ | 83.92 | 86.68 | 77.05 | 79.11 | 85.20 | 88.59 | 78.23 | 78.64 | **3.34 (1.12-5.56)** | **2.79 (0.21-5.36)** | 4.73 (-0.31-9.77) | 4.62 (-2.64-11.88) |
| *Confidence in vaccine efficacy (maternal influenza vaccine)^1^* | 76.46 | 83.86 | 55.90 | 67.33 | 79.93 | 87.14 | 63.48 | 68.75 | **4.09 (1.87-6.31)** | **3.86 (1.59-6.13)** | **6.81 (0.83-12.80)** | 3.04 (-4.25-10.34) |
| Getting the flu vaccine will reduce (reduced/would have reduced) my risk of getting the flu during my pregnancy^1^ | 78.42 | 86.67 | 56.79 | 66.22 | 80.75 | 88.85 | 64.18 | 66.59 | 2.24 (-0.11-4.58) | 2.03 (-0.35-4.40) | 6.22 (-0.27-12.71) | 0.38 (-7.76-8.53) |
| Getting the flu vaccine while I am pregnant will reduce (reduced/would have reduced) my baby’s risk of getting the flu^1^ | 74.69 | 81.37 | 55.0 | 68.44 | 79.54 | 86.26 | 63.18 | 70.91 | **6.21 (3.68-8.74)** | **5.64 (2.95-8.33)** | **8.91 (2.55-15.27)** | 6.06 (-1.35-13.47) |
| *Perceived risk (maternal whooping cough)* |  |  |  |  |  |  |  |  |  |  |  |  |
| Whooping cough is dangerous for pregnant women^2^ | 83.94 | 85.69 | 78.89 | 81.11 | 84.88 | 87.03 | 80.28 | 79.77 | 0.73 (-1.41-2.86) | 0.44 (-2.05-2.94) | 0.33 (-4.81-5.48) | 3.35 (-3.40-10.09) |
| *Perceived risk (infant whooping cough)^4^* | 81.19 | 83.00 | 73.42 | 70.62 | 81.47 | 82.71 | 73.33 | 77.44 | 0.07 (-1.41-1.54) | -0.27 (-1.82-1.28) | -1.24 (-6.59-4.11) | **6.58 (0.01-13.15)** |
| I worry that my baby could get whooping cough^4^ | 60.94 | 63.48 | 49.87 | 47.04 | 59.30 | 60.90 | 49.85 | 53.46 | -2.11 (-4.97-0.75) | -2.72 (-5.84-0.41) | -3.09 (-11.73-5.55) | 7.02 (-4.40-18.43) |
| Whooping cough is dangerous for babies^4^ | 91.93 | 93.36 | 86.08 | 82.96 | 93.49 | 94.55 | 86.06 | 90.00 | 1.54 (-0.05-3.14) | 1.17 (-0.47-2.82) | 0.60 (-5.72-6.92) | **7.68 (0.46-14.90)** |
| Whooping cough is more dangerous for babies than older children or adults^4^ | 91.07 | 92.63 | 84.30 | 81.85 | 91.89 | 92.82 | 85.37 | 88.85 | 0.43 (-1.30-2.16) | 0.14 (-1.66-1.93) | 0.58 (-5.58-6.74) | 5.92 (-2.79-14.63) |
| *Confidence in vaccine efficacy (whooping cough vaccine)^2^* | 83.86 | 88.69 | 69.24 | 77.33 | 86.27 | 90.66 | 74.65 | 79.38 | 0.17 (-1.57-1.92) | 0.09 (-1.72-1.90) | -0.08 (-5.24-5.07) | 1.79 (-3.77-7.35) |
| Whooping cough vaccine will reduce (reduced/would have reduced) my chances of getting whooping cough^2^ | 82.36 | 87.55 | 66.53 | 75.56 | 84.44 | 88.38 | 74.65 | 77.27 | 0.57 (-1.59-2.73) | -0.33 (-2.61-1.96) | 3.54 (-2.63-9.72) | 0.05 (-6.93-7.03) |
| Whooping cough vaccine will reduce (reduced/would have reduced) the chance of me giving whooping cough to my baby^2^ | 84.64 | 89.62 | 69.03 | 78.89 | 87.33 | 91.95 | 75.35 | 79.77 | 0.39 (-1.17-2.48) | 0.72 (-1.43-2.88) | 2.17 (-4.20-8.54) | 0.36 (-5.32-6.04) |
| Getting the whooping cough vaccine while I am pregnant will reduce (reduced/would have reduced) my baby’s risk of getting whooping cough^2^ | 83.20 | 88.23 | 68.19 | 75.96 | 86.60 | 91.19 | 74.71 | 78.86 | 1.25 (-0.93-3.43) | 1.26 (-1.01-3.52) | 1.95 (-4.37-8.27) | 1.21 (-5.62-8.04) |
| Getting the whooping cough vaccine for my baby will reduce my baby’s chances of getting whooping cough^4^ | 86.10 | 89.41 | 69.62 | 70.00 | 87.62 | 90.99 | 63.58 | 78.46 | 0.24 (-1.73-2.22) | 0.26 (-1.62-2.14) | -3.69 (-12.56-5.18) | 5.07 (-4.39-14.52) |
| *Belief in superiority of natural immunity^d^* |  |  |  |  |  |  |  |  |  |  |  |  |
| I believe it is better for my baby to develop their own immunity by getting sick rather than by getting a vaccine^4d^ | 43.39 | 40.28 | 58.99 | 58.89 | 44.49 | 41.03 | 64.85 | 58.43 | 0.33 (-2.60-3.26) | 0.45 (-2.68-3.57) | -2.74 (-13.14-7.67) | -0.32 (-11.61-10.97) |
| *Pro-vaccine social norms^4^* | 83.16 | 86.12 | 70.00 | 67.59 | 84.01 | 86.67 | 66.06 | 74.71 | 0.37 (-1.08-1.82) | 0.43 (-0.99-1.84) | -4.75 (-11.17-1.68) | 4.78 (-2.44-12.00) |
| The majority of my friends and family would get all of the vaccines recommended for their babies after the birth^4^ | 83.49 | 86.37 | 70.63 | 68.52 | 84.55 | 87.04 | 68.18 | 75.29 | 0.65 (-0.91-2.21) | 0.61 (-0.92-2.14) | -3.20 (-10.07-3.68) | 4.10 (-3.43-11.63) |
| The majority of my friends and family would encourage me to get all of the vaccines recommended for my baby^4^ | 82.93 | 86.01 | 69.37 | 66.67 | 83.70 | 86.57 | 63.94 | 74.12 | 0.27 (-1.31-1.86) | 0.41 (-1.12-1.94) | -6.21 (-13.18-0.75) | 5.84 (-2.42-14.09) |
| *Self-efficacy* |  |  |  |  |  |  |  |  |  |  |  |  |
| It is in my control whether or not my baby gets his/her vaccines^4^ | 91.41 | 92.52 | 89.37 | 81.48 | 92.00 | 92.66 | 88.18 | 89.02 | 1.19 (-0.48-2.86) | 0.74 (-0.89-2.37) | -0.66 (-8.12-6.80) | **9.67 (0.65-18.69)** |
| *Perceived vaccine knowledge^4^* | 84.45 | 86.37 | 74.18 | 75.74 | 86.46 | 88.28 | 78.64 | 74.42 | **2.37 (0.93-3.80)** | **2.42 (1.01-3.82)** | 4.68 (-1.17-10.53) | -2.40 (-10.69-5.89) |
| I have most of the important information I need to make a decision about vaccines for my baby^4^ | 86.14 | 88.06 | 75.95 | 77.41 | 87.82 | 89.61 | 80.61 | 74.90 | **1.98 (0.50-3.47)** | **1.92 (0.49-3.35)** | 5.27 (-1.34-11.89) | -2.35 (-10.84-6.14) |
| I know enough about the safety of the whooping cough vaccine to make a decision about getting the vaccine for my baby^4^ | 82.96 | 84.94 | 72.41 | 74.07 | 85.46 | 87.23 | 76.67 | 75.38 | **2.80 (1.07-4.52)** | **2.78 (1.07-4.50)** | 4.55 (-2.79-11.88) | -0.75 (-9.71-8.22) |
| *Trust in vaccine information (from obstetricians and pediatricians)^4^* | 87.32 | 90.49 | 70.38 | 73.89 | 88.76 | 76.35 | 70.15 | 76.35 | 1.29 (-0.04-2.62) | 1.12 (-0.01-2.25) | 1.72 (-4.86-8.29) | 1.88 (-5.50-9.25) |
| I trust the information provided by my obstetrician or midwife about vaccines during pregnancy^3^ | 87.96 | 90.35 | 76.77 | 83.60 | 89.32 | 92.25 | 76.63 | 82.30 | 1.32 (-0.07-2.72) | **1.54 (0.18-2.91)** | 1.11 (-4.73-6.96) | -0.32 (-5.32-4.67) |
| I trust the information provided by my obstetrician or midwife about vaccines for babies^4^ | 86.69 | 89.71 | 71.79 | 71.85 | 88.64 | 91.66 | 70.00 | 76.15 | **1.92 (0.49-3.35)** | **1.70 (0.41-2.98)** | 2.32 (-4.52-9.17) | 3.66 (-4.64-11.95) |
| I trust the information provided by my baby's doctor about vaccines during pregnancy^3^ | 87.78 | 90.43 | 76.34 | 81.84 | 88.72 | 91.66 | 75.66 | 82.07 | 0.50 (-0.83-1.83) | 0.64 (-0.62-1.91) | 0.53 (-5.84-6.91) | -0.11 (-4.72-4.50) |
| I trust the information provided by my baby's doctor about vaccines for babies^4^ | 88.55 | 91.70 | 71.20 | 74.81 | 89.43 | 92.42 | 71.88 | 75.38 | 0.49 (-0.82-1.80) | 0.34 (-0.80-1.48) | 5.32 (-1.67-12.32) | -2.74 (-11.48-6.00) |
| *Trust in vaccine information (from naturopaths and chiropractors)^4^* | 67.16 | 66.74 | 69.20 | 68.38 | 70.82 | 70.71 | 70.45 | 71.16 | 0.61 (-2.62-3.84) | -0.16 (-3.76-3.45) | 1.92 (-8.26-12.11) | 0.09 (-10.49-10.66) |
| I trust the information provided by naturopathic and/or chiropractic doctors about vaccines during pregnancy^3^ | 71.44 | 71.10 | 76.72 | 66.67 | 75.16 | 75.33 | 76.25 | 72.96 | 1.42 (-1.46-4.29) | 2.02 (-1.37-5.42) | -0.43 (-7.44-6.59) | 1.61 (-7.08-10.31) |
| I trust the information provided by naturopathic and/or chiropractic doctors about vaccines for babies^4^ | 70.58 | 70.17 | 71.91 | 72.73 | 74.62 | 74.62 | 74.50 | 74.00 | 0.73 (-2.19-3.64) | 0.97 (-2.24-4.18) | -0.47 (-10.40-9.46) | -2.45 (-12.44-7.55) |
| *Trust in vaccine information (from federal agencies and academic institutions)^4^* | 83.84 | 87.29 | 65.77 | 68.52 | 83.94 | 87.43 | 60.08 | 72.12 | 0.40 (-0.90-1.70) | 0.39 (-0.82-1.60) | -2.69 (-9.00-3.62) | 2.47 (-4.43-9.38) |
| I trust the information provided by federal agencies such as the Centers for Disease Control and Prevention (CDC) about vaccines during pregnancy^3^ | 83.29 | 86.81 | 66.24 | 77.27 | 83.71 | 87.86 | 62.20 | 76.78 | 0.67 (-0.88-2.23) | 1.02 (-0.52-2.55) | -2.39 (-8.99-4.21) | 0.86 (-4.67-6.38) |
| I trust the information provided by federal agencies such as the Centers for Disease Control and Prevention (CDC) about vaccines for babies^4^ | 83.12 | 87.12 | 62.82 | 64.44 | 83.64 | 87.89 | 54.24 | 70.00 | 0.72 (-0.83-2.28) | 0.94 (-0.47-2.36 | -4.33 (-11.82-3.16) | 3.70 (-5.19-12.59) |
| I trust the information provided by scientists and doctors at universities and academic institutions about vaccines during pregnancy^3^ | 84.52 | 87.32 | 70.97 | 79.77 | 84.29 | 87.40 | 67.62 | 80.00 | 0.05 (-1.40-1.49) | 0.27 (-1.17-1.70) | -2.84 (-8.83-3.15) | 0.25 (-4.44-4.93) |
| I trust the information provided by scientists and doctors at universities and academic institutions about vaccines for babies^4^ | 84.64 | 87.48 | 68.72 | 74.07 | 84.46 | 87.23 | 66.67 | 73.46 | 0.06 (-1.35-1.46) | 0.02 (-1.35-1.38) | -0.54 (-7.30-6.21) | -1.49 (-7.89-4.91) |

^a^Constructs are italicized, with constituent statements listed below. For constructs and statements specific to maternal influenza disease or vaccine, "intend to vaccinate", "intend not to vaccinate", and "uncertain intentions" refer to maternal influenza vaccine^1^; for constructs and statements specific to maternal pertussis disease or vaccine, "intend to vaccinate", "intend not to vaccinate", and "uncertain intentions" refer to maternal Tdap vaccine^2^; for constructs and statements relevant to both maternal influenza and pertussis diseases/vaccines, "intend to vaccinate", "intend not to vaccinate", and "uncertain intentions" refer to at least one of maternal influenza and Tdap vaccines^3^; for constructs and statements specific to infant diseases or vaccines, "intend to vaccinate", "intend not to vaccinate", and "uncertain intentions" refer to intending to receive all recommended infant vaccines versus intending to receive some or no recommended infant vaccines^4^; for constructs and statements relevant to both maternal and infant vaccines, "intend to vaccinate", "intend not to vaccinate", and "uncertain intentions" refer to intending to receive all recommended infant vaccines versus intending to receive some or no recommended infant vaccines^4^; and for constructs with constituent statements belonging to different of the above categories, "intend to vaccinate", "intend not to vaccinate", and "uncertain intentions" refer to the most common category, and in the case of a tie refer to intending to receive all recommended infant vaccines versus intending to receive some or no recommended infant vaccines^4^.

^b^RC=Regression Coefficient (from linear regression); 95%CI = 95% Confidence Interval; bolded if statistically significant.

^c^KAB = knowledge, attitudes, and beliefs. Likert scale responses were encoded as follows: 1 - strongly disagree, 2 - disagree, 3 - don’t know (only included for items assessing knowledge or trust), 4 - agree, 5 - strongly agree. Construct summary scores were created for all constructs with multiple constituent survey statements at each timepoint. All continuous statement and construct scores were standardized by dividing the maximum score within the sample then multiplying by 100.

^d^Negatively phrased construct or statement, for which a negative association would indicate a positive effect on vaccine KAB (other constructs and statements were created from positively phrased survey statements, for which a positive association would indicate a positive effect on vaccine KAB)

**Appendix 4. Impact of MomsTalkShots on Vaccine KABs of Women One Year After Their Infant's Birth**

individual survey items, organized by constructs, stratified by baseline vaccine intentions^a^, continuous analysis^b^

|  | Average scaled scores^c^ among those **not** receiving MomsTalkShots | | | | Average scaled scores^c^ among those receiving MomsTalkShots | | | | Effect of MomsTalkShots on average scaled scores^c^, RC (95% CI)** | | | |
| --- | --- | --- | --- | --- | --- | --- | --- | --- | --- | --- | --- | --- |
| *Intentions to Vaccinate^a^* | *All* | *Yes* | *No* | *Unsure* | *All* | *Yes* | *No* | *Unsure* | *All* | *Yes* | *No* | *Unsure* |
| ***KAB Constructs* and their Constituent Statements^c^** |  |  |  |  |  |  |  |  |  |  |  |  |
| *Confidence in vaccine safety (for the infant)* |  |  |  |  |  |  |  |  |  |  |  |  |
| I am confident that vaccines are safe for my baby^4^ | 86.27 | 90.10 | 64.83 | 67.88 | 87.62 | 91.35 | 57.62 | 72.35 | 0.79 (-0.91-2.50) | 0.74 (-0.86-2.34) | -3.45 (-11.87-4.97) | 7.68 (-2.66-18.01) |
| *Specific safety concerns (for infant vaccines)^4d^* | 32.90 | 29.48 | 52.93 | 47.73 | 31.84 | 28.31 | 60.12 | 46.47 | -0.87 (-2.43-0.69) | -0.83 (-2.28-0.62) | 1.76 (-6.56-10.09) | -4.62 (-13.87-4.64) |
| It is better for babies to get fewer vaccines at the same time^4d^ | 33.41 | 30.06 | 53.10 | 47.88 | 32.38 | 28.78 | 60.95 | 47.65 | -0.56 (-2.35-1.22) | -0.99 (-2.66-0.67) | 5.78 (-3.46-15.02) | -1.14 (-12.62-10.35) |
| Babies get more vaccines in their first two years of life than are good for them^4d^ | 32.96 | 29.48 | 53.45 | 47.88 | 31.83 | 28.27 | 59.52 | 47.65 | -1.03 (-2.75-0.69) | -0.88 (-2.39-0.62) | 1.58 (-8.70-11.87) | -2.97 (-13.44-7.49) |
| Vaccines often cause serious side effects in babies^4d^ | 32.30 | 29.15 | 51.72 | 44.24 | 31.07 | 27.93 | 56.67 | 43.53 | -1.20 (-2.76-0.37) | -0.86 (-2.27-0.55) | 0.87 (-7.74-9.48) | -3.57 (-13.23-6.10) |
| The ingredients in vaccines are not safe for my baby^4d^ | 32.93 | 29.23 | 53.45 | 50.91 | 32.09 | 28.27 | 63.33 | 47.06 | -0.76 (-2.36-0.84) | -0.64 (-02.10-0.81) | 1.67 (-6.84-10.17) | -6.75 (-16.86-3.36) |
| *Perceived risk (infant whooping cough)^4^* | 82.41 | 83.81 | 74.60 | 74.75 | 81.16 | 81.79 | 73.17 | 81.76 | 0.10 (-1.66-1.86) | -0.16 (-2.03-1.71) | -1.47 (-8.83-5.89) | 2.70 (-4.04-9.44) |
| I worry that my baby could get whooping cough^4^ | 62.36 | 63.85 | 52.76 | 55.76 | 58.04 | 58.44 | 50.00 | 61.76 | -2.25 (-5.87-1.37) | -2.28 (-6.25-1.69) | -1.25 (-12.82-10.33) | -4.66 (-18.60-9.27) |
| Whooping cough is dangerous for babies^4^ | 93.70 | 95.09 | 86.90 | 84.85 | 93.77 | 94.40 | 86.67 | 93.53 | 0.43 (-1.47-2.33) | -0.29 (-2.27-1.70) | -0.43 (-8.71-7.85) | **8.52 (1.39-15.65)** |
| Whooping cough is more dangerous for babies than older children or adults^4^ | 91.38 | 92.74 | 84.14 | 83.64 | 92.27 | 93.25 | 82.86 | 90.00 | **2.20 (0.23-4.17)** | **2.11 (0.11-4.10)** | -2.61 (-12.43-7.21) | 4.92 (-3.48-13.32) |
| *Confidence in vaccine efficacy (whooping cough vaccine)* |  |  |  |  |  |  |  |  |  |  |  |  |
| Getting the whooping cough vaccine for my baby will reduce (reduced/would have reduced) my baby’s chances of getting whooping cough^4^ | 88.87 | 91.86 | 72.76 | 72.73 | 89.46 | 92.00 | 67.62 | 81.18 | 0.39 (-1.76-2.55) | 0.49 (-1.47-2.46) | -3.26 (-13.23-6.72) | -1.08 (-13.98-11.81) |
| *Belief in superiority of natural immunity^d^* |  |  |  |  |  |  |  |  |  |  |  |  |
| I believe it is better for my baby to develop their own immunity by getting sick rather than by getting a vaccine^4d^ | 39.44 | 36.97 | 54.14 | 50.91 | 40.83 | 38.48 | 58.10 | 52.35 | 1.05 (-2.28-4.39) | 1.94 (-1.63-5.52) | -0.27 (-12.72-12.17) | -6.05 (-18.19-6.09) |
| *Pro-vaccine social norms^4^* | 84.30 | 86.57 | 71.38 | 75.15 | 86.68 | 89.03 | 68.33 | 76.76 | **1.86 (0.13-3.59)** | **2.38 (0.67-4.10)** | -5.63 (-13.84-2.59) | -0.37 (-9.33-8.58) |
| The majority of my friends and family would get all of the vaccines recommended for their babies after the birth^4^ | 85.48 | 87.44 | 73.45 | 79.39 | 87.57 | 89.56 | 72.38 | 78.82 | 1.54 (-0.21-3.30) | **1.87 (0.11-3.63)** | -2.77 (-10.83-5.29) | -1.58 (-11.06-7.90) |
| The majority of my friends and family would encourage me to get all of the vaccines recommended for my baby^4^ | 83.55 | 86.24 | 69.31 | 70.91 | 85.80 | 88.51 | 64.29 | 74.71 | 1.82 (-0.13-3.78) | **2.34 (0.44-4.25)** | -7.46 (-16.99-2.06) | 1.81 (-8.05-11.66) |
| *Self-efficacy* |  |  |  |  |  |  |  |  |  |  |  |  |
| It is in my control whether or not my baby gets his/her vaccines^4^ | 89.98 | 91.61 | 86.21 | 73.94 | 92.57 | 92.93 | 89.52 | 91.76 | 2.05 (-0.11-4.20) | 0.84 (-1.30-2.97) | 2.54 (-6.57-11.64) | **17.91 (5.72-28.67)** |
| *Perceived vaccine knowledge^4^* | 85.36 | 87.13 | 79.14 | 69.70 | 89.20 | 90.06 | 84.76 | 83.24 | **4.16 (2.58-5.74)** | **3.37 (1.74-5.00)** | 5.44 (-0.83-11.72) | **12.78 (4.17-21.39)** |
| I have most of the important information I need to make a decision about vaccines for my baby^4^ | 86.72 | 88.44 | 81.03 | 71.52 | 90.67 | 91.49 | 86.19 | 84.24 | **4.05 (2.43-5.67)** | **3.22 (1.57-4.87)** | 4.82 (-1.80-11.43) | **12.42 (3.46-21.38)** |
| I know enough about the safety of the whooping cough vaccine to make a decision about getting the vaccine for my baby^4^ | 84.44 | 86.36 | 77.24 | 67.88 | 87.90 | 88.63 | 83.33 | 84.71 | **3.89 (2.04-5.75)** | **2.85 (0.95-4.75)** | 6.19 (-1.49-13.86) | **16.24 (6.05-26.43)** |
| *Trust in vaccine information (from obstetricians and pediatricians)^4^* | 87.56 | 90.36 | 70.26 | 76.21 | 90.35 | 92.86 | 68.57 | 82.50 | **2.98 (1.44-4.52)** | **2.61 (1.20-4.01)** | 1.49 (-6.03-9.01) | 5.70 (-2.20-13.60) |
| I trust the information provided by my obstetrician or midwife about vaccines during pregnancy^3^ | 88.24 | 91.12 | 72.75 | 84.21 | 90.53 | 92.67 | 78.57 | 86.56 | **2.36 (0.83-3.89)** | 1.33 (-0.09-2.75) | **7.58 (0.44-14.72)** | 3.26 (-2.30-8.81) |
| I trust the information provided by my obstetrician or midwife about vaccines for babies^4^ | 87.27 | 90.17 | 69.31 | 75.76 | 89.69 | 92.00 | 68.10 | 84.24 | **2.65 (1.04-4.25)** | **1.83 (0.30-3.35)** | 4.32 (-3.82-12.46) | **7.97 (0.54-15.39)** |
| I trust the information provided by my baby's doctor about vaccines during pregnancy^3^ | 87.83 | 90.09 | 73.82 | 86.67 | 90.42 | 92.84 | 77.89 | 85.31 | **2.08 (0.54-3.63)** | **2.09 (0.55-3.64)** | 6.36 (-0.31-13.03) | -3.59 (-9.03-1.85) |
| I trust the information provided by my baby's doctor about vaccines for babies^4^ | 88.59 | 91.45 | 72.07 | 75.15 | 91.27 | 93.68 | 69.52 | 84.71 | **2.25 (0.74-3.76)** | **1.98 (0.58-3.38)** | 0.31 (-7.41-8.04) | 8.04 (-1.58-17.66) |
| *Trust in vaccine information (from naturopaths and chiropractors)^4^* | 65.41 | 64.11 | 68.81 | 72.17 | 68.14 | 68.12 | 67.00 | 69.63 | 0.82 (-3.16-4.80) | 1.16 (-3.38-5.71) | -2.34 (-13.65-8.97) | -1.58 (-13.68-10.52) |
| I trust the information provided by naturopathic and/or chiropractic doctors about vaccines during pregnancy^3^ | 69.22 | 67.03 | 72.14 | 77.14 | 73.45 | 72.06 | 74.29 | 80.00 | 2.42 (-1.21-6.06) | 1.83 (-2.51-6.16) | 5.20 (-3.23-13.63) | -0.20 (-9.79-9.40) |
| I trust the information provided by naturopathic and/or chiropractic doctors about vaccines for babies^4^ | 67.55 | 66.23 | 71.28 | 73.64 | 72.00 | 71.86 | 71.11 | 74.17 | 1.80 (-2.12-5.72) | 1.41 (-3.08-5.91) | 2.79 (-7.40-12.98) | 0.90 (-11.31-13.11) |
| *Trust in vaccine information (from federal agencies and academic institutions)^4^* | 84.88 | 88.00 | 66.81 | 70.61 | 86.25 | 88.89 | 64.40 | 76.18 | **1.97 (0.43-3.51)** | 1.39 (-0.12-2.89) | 3.05 (-4.32-10.42) | 3.60 (-4.77-11.96) |
| I trust the information provided by federal agencies such as the Centers for Disease Control and Prevention (CDC) about vaccines during pregnancy^3^ | 85.11 | 88.20 | 66.18 | 83.16 | 86.05 | 89.47 | 67.02 | 80.00 | 1.29 (-0.47-3.05) | 1.53 (-0.17-3.23) | -0.45 (-8.64-7.73) | -1.22 (-7.76-5.32) |
| I trust the information provided by federal agencies such as the Centers for Disease Control and Prevention (CDC) about vaccines for babies^4^ | 84.65 | 88.45 | 63.10 | 66.67 | 86.01 | 89.22 | 58.57 | 75.29 | 1.67 (-0.08-3.43) | 1.25 (-0.42-2.91) | 1.85 (-6.97-10.66) | 4.92 (-5.40-15.23) |
| I trust the information provided by scientists and doctors at universities and academic institutions about vaccines during pregnancy^3^ | 85.29 | 87.57 | 71.18 | 83.86 | 86.41 | 88.31 | 76.14 | 82.81 | **1.81 (0.08-3.54)** | 1.19 (-0.48-2.87) | 6.47 (-1.88-14.83) | -0.70 (-6.44-5.03) |
| I trust the information provided by scientists and doctors at universities and academic institutions about vaccines for babies^4^ | 85.50 | 88.17 | 69.66 | 73.94 | 86.52 | 88.72 | 69.52 | 76.47 | 1.66 (-0.03-3.34) | 0.94 (-0.72-2.60) | 4.52 (-3.64-12.67) | 1.28 (-6.87-9.42) |

^a^Constructs are italicized, with constituent statements listed below. For constructs and statements specific to maternal influenza disease or vaccine, "intend to vaccinate", "intend not to vaccinate", and "uncertain intentions" refer to maternal influenza vaccine^1^; for constructs and statements specific to maternal pertussis disease or vaccine, "intend to vaccinate", "intend not to vaccinate", and "uncertain intentions" refer to maternal Tdap vaccine^2^; for constructs and statements relevant to both maternal influenza and pertussis diseases/vaccines, "intend to vaccinate", "intend not to vaccinate", and "uncertain intentions" refer to at least one of maternal influenza and Tdap vaccines^3^; for constructs and statements specific to infant diseases or vaccines, "intend to vaccinate", "intend not to vaccinate", and "uncertain intentions" refer to intending to receive all recommended infant vaccines versus intending to receive some or no recommended infant vaccines^4^; for constructs and statements relevant to both maternal and infant vaccines, "intend to vaccinate", "intend not to vaccinate", and "uncertain intentions" refer to intending to receive all recommended infant vaccines versus intending to receive some or no recommended infant vaccines^4^; and for constructs with constituent statements belonging to different of the above categories, "intend to vaccinate", "intend not to vaccinate", and "uncertain intentions" refer to the most common category, and in the case of a tie refer to intending to receive all recommended infant vaccines versus intending to receive some or no recommended infant vaccines^4^.

^b^RC=Regression Coefficient (from linear regression); 95%CI = 95% Confidence Interval; bolded if statistically significant.

^c^KAB = knowledge, attitudes, and beliefs. Likert scale responses were encoded as follows: 1 - strongly disagree, 2 - disagree, 3 - don’t know (only included for items assessing knowledge or trust), 4 - agree, 5 - strongly agree. Construct summary scores were created for all constructs with multiple constituent survey statements at each timepoint. All continuous statement and construct scores were standardized by dividing the maximum score within the sample then multiplying by 100.

^d^Negatively phrased construct or statement, for which a negative association would indicate a positive effect on vaccine KAB (other constructs and statements were created from positively phrased survey statements, for which a positive association would indicate a positive effect on vaccine KAB)

**Appendix 5. Impact of MomsTalkShots on Women's Perceived Risk of Infant Pertussis One Month After Their Infant's Birth**

individual survey items, stratified by baseline vaccine intentions^a^ and Tdap vaccination, dichotomous analysis^b^

|  | % agreeing with statements or with above average construct scores^c^ among those **not** receiving MomsTalkShots | | | | % agreeing with statements or with above average construct scores^c^ among those receiving MomsTalkShots | | | | Effect of MomsTalkShots on % agreeing with statements or with above average construct scores^c^,  OR (95% CI)^b^ | | | |
| --- | --- | --- | --- | --- | --- | --- | --- | --- | --- | --- | --- | --- |
| *Intentions to Vaccinate^a^* | *All* | *Yes* | *No* | *Unsure* | *All* | *Yes* | *No* | *Unsure* | *All* | *Yes* | *No* | *Unsure* |
| **All** |  |  |  |  |  |  |  |  |  |  |  |  |
| *Perceived risk (infant whooping cough)* | 42 | 47 | 22 | 19 | 39 | 42 | 19 | 31 | **0.75 (0.57-1.00)^e^** | **0.70 (0.51-0.96)** | 0.77 (0.27-2.24) | 2.18 (0.68-6.96) |
| I worry that my baby could get whooping cough | 51 | 55 | 30 | 24 | 47 | 49 | 33 | 37 | 0.78 (0.59-1.03) | **0.72 (0.53-0.98)** | 0.78 (0.31-2.01) | 1.98 (0.67-5.85) |
| Whooping cough is dangerous for babies | 96 | 97 | 90 | 98 | 98 | 98 | 98 | 85 | **2.69 (1.14-6.37)** | 1.59 (0.61-4.15) | 3.83 (0.45-32.72) | N/A^d^ |
| Whooping cough is more dangerous for babies than older children or adults | 93 | 95 | 86 | 81 | 94 | 94 | 91 | 90 | 1.15 (0.68-1.93) | 0.86 (0.47-1.57) | 3.19 (0.63-16.22) | 2.26 (0.44-11.51) |
| **Received maternal Tdap** |  |  |  |  |  |  |  |  |  |  |  |  |
| *Perceived risk (infant whooping cough)* | 42 | 48 | 26 | 21 | 39 | 42 | 19 | 33 | **0.72 (0.53-0.98)** | **0.68 (0.49-0.94)** | 0.64 (0.14-2.87) | 1.83 (0.41-8.10) |
| I worry that my baby could get whooping cough | 54 | 57 | 33 | 29 | 48 | 50 | 34 | 39 | **0.72 (0.53-0.97)** | **0.67 (0.49-0.92)** | 0.67 (0.17-2.63) | 1.67 (0.42-6.65) |
| Whooping cough is dangerous for babies | 96 | 97 | 93 | 88 | 99 | 99 | 100 | 97 | 2.69 (0.90-8.02) | 1.99 (0.64-6.18) | N/A^d^ | N/A^d^ |
| Whooping cough is more dangerous for babies than older children or adults | 94 | 95 | 91 | 82 | 95 | 95 | 94 | 92 | 1.09 (0.58-2.07) | 0.90 (0.45-1.78) | 1.33 (0.12-14.60) | N/A^d^ |
| **Did not receive maternal Tdap** |  |  |  |  |  |  |  |  |  |  |  |  |
| *Perceived risk (infant whooping cough)* | 42 | 38 | 17 | 15 | 39 | 40 | 20 | 25 | 1.03 (0.48-2.23) | 1.21 (0.42-3.49) | 0.24 (0.01-4.08) | N/A^d^ |
| I worry that my baby could get whooping cough | 35 | 43 | 28 | 15 | 41 | 48 | 31 | 31 | 1.40 (0.65-3.03) | 2.17 (0.68-6.94) | 0.77 (0.16-3.59) | 3.07 (0.44-21.37) |
| Whooping cough is dangerous for babies | 92 | 97 | 86 | 80 | 95 | 93 | 97 | 100 | 3.09 (0.70-13.74) | 0.74 (0.10-5.53) | 4.71 (0.39-57.09) | N/A^d^ |
| Whooping cough is more dangerous for babies than older children or adults | 88 | 93 | 81 | 80 | 87 | 87 | 89 | 88 | 1.16 (0.44-3.07) | 0.96 (0.19-4.87) | 5.75 (0.57-57.60) | 1.18 (0.13-11.11) |

^a^As the perceived risk (infant whooping cough) construct (in italics) and its constituent statements (listed below the construct) are specific to infant disease, "intend to vaccinate", "intend not to vaccinate", and "uncertain intentions" refer to intending to receive all recommended infant vaccines versus intending to receive some or no recommended infant vaccines.

^b^OR = Odds Ratio (from logistic regression); 95%CI = 95% Confidence Interval; bolded if statistically significant.

^c^KAB = knowledge, attitudes, and beliefs. Likert scale responses were encoded as follows: 1 - strongly disagree, 2 - disagree, 3 - don’t know (only included for items assessing knowledge or trust), 4 - agree, 5 - strongly agree. Summary scores were created for all KAB constructs with multiple constituent survey statements at each timepoint. Dichotomous variables assessing construct summary scores coded scores above the average as 1 and scores below the average as 0.

^d^N/A = not applicable (n=1 in a cell of 2-by-2 table, p>0.99; and/or no convergence achieved)

^e^This appearance of an overlap with 1 in the 95%CI is due to rounding

**Appendix 6. Impact of MomsTalkShots on Women's Perceived Risk of Infant Pertussis One Year After Their Infant's Birth**

individual survey items, stratified by baseline vaccine intentions^a^ and Tdap vaccination, dichotomous analysis^b^

|  | % agreeing with statements or with above average construct scores^c^ among those **not** receiving MomsTalkShots | | | | % agreeing with statements or with above average construct scores^c^ among those receiving MomsTalkShots | | | | Effect of MomsTalkShots on % agreeing with statements or with above average construct scores^c^,  OR (95% CI)^b^ | | | |
| --- | --- | --- | --- | --- | --- | --- | --- | --- | --- | --- | --- | --- |
| *Intentions to Vaccinate^a^* | *All* | *Yes* | *No* | *Unsure* | *All* | *Yes* | *No* | *Unsure* | *All* | *Yes* | *No* | *Unsure* |
| **All** |  |  |  |  |  |  |  |  |  |  |  |  |
| *Perceived risk (infant whooping cough)* | 46 | 49 | 26 | 24 | 39 | 40 | 19 | 44 | 0.89 (0.66-1.21) | 0.89 (0.64-1.22) | 0.53 (0.13-2.27) | 1.05 (0.27-4.08) |
| I worry that my baby could get whooping cough | 55 | 57 | 38 | 42 | 46 | 47 | 31 | 50 | 0.79 (0.58-1.08) | 0.80 (0.58-1.11) | 0.85 (0.25-2.91) | 0.50 (0.13-1.92) |
| Whooping cough is dangerous for babies | 96 | 98 | 88 | 88 | 97 | 97 | 95 | 97 | 1.37 (0.60-3.13) | 0.78 (0.30-2.04) | 3.04 (0.32-28.72) | N/A^d^ |
| Whooping cough is more dangerous for babies than older children or adults | 93 | 94 | 79 | 88 | 95 | 96 | 83 | 97 | **1.98 (1.02-3.87)** | 1.96 (0.86-4.47) | 1.18 (0.31-4.40) | N/A^d^ |
| **Received maternal Tdap** |  |  |  |  |  |  |  |  |  |  |  |  |
| *Perceived risk (infant whooping cough)* | 46 | 51 | 37 | 21 | 39 | 41 | 13 | 43 | 0.84 (0.60-1.16) | 0.86 (0.61-1.22) | N/A^d^ | 0.47 (0.04-5.33) |
| I worry that my baby could get whooping cough | 58 | 59 | 47 | 46 | 47 | 47 | 33 | 48 | 0.73 (0.52-1.01) | 0.74 (0.53-1.05) | 0.44 (0.07-2.95) | 0.31 (0.04-2.48) |
| Whooping cough is dangerous for babies | 97 | 98 | 100 | 83 | 97 | 97 | 100 | 96 | 0.98 (0.36-2.65) | N/A^d^ | N/A^d^ | N/A^d^ |
| Whooping cough is more dangerous for babies than older children or adults | 93 | 94 | 83 | 92 | 96 | 96 | 93 | 96 | 2.11 (0.94-4.74) | 2.02 (0.84-4.89) | 1.11 (0.10-11.84) | N/A^d^ |
| **Did not receive maternal Tdap** |  |  |  |  |  |  |  |  |  |  |  |  |
| *Perceived risk (infant whooping cough)* | 46 | 40 | 14 | 33 | 39 | 33 | 22 | 45 | 1.33 (0.60-2.94) | 1.22 (0.43-3.46) | 1.98 (0.33-11.75) | N/A^d^ |
| I worry that my baby could get whooping cough | 41 | 46 | 29 | 33 | 42 | 46 | 30 | 55 | 1.30 (0.57-2.94) | 1.61 (0.57-4.53) | 1.51 (0.18-12.44) | 1.13 (0.11-11.25) |
| Whooping cough is dangerous for babies | 91 | 96 | 75 | 100 | 96 | 98 | 93 | 100 | 2.91 (0.57-14.8) | 1.17 (0.11-12.4) | 4.82 (0.44-53.09) | N/A^d^ |
| Whooping cough is more dangerous for babies than older children or adults | 89 | 96 | 75 | 78 | 90 | 95 | 78 | 100 | 1.89 (0.53-6.65) | 1.61 (0.14-18.75) | 1.25 (0.24-6.41) | N/A^d^ |

^a^As the perceived risk (infant whooping cough) construct (in italics) and its constituent statements (listed below the construct) are specific to infant disease, "intend to vaccinate", "intend not to vaccinate", and "uncertain intentions" refer to intending to receive all recommended infant vaccines versus intending to receive some or no recommended infant vaccines.

^b^OR = Odds Ratio (from logistic regression); 95%CI = 95% Confidence Interval; bolded if statistically significant.

^c^KAB = knowledge, attitudes, and beliefs. Likert scale responses were encoded as follows: 1 - strongly disagree, 2 - disagree, 3 - don’t know (only included for items assessing knowledge or trust), 4 - agree, 5 - strongly agree. Summary scores were created for all KAB constructs with multiple constituent survey statements at each timepoint. Dichotomous variables assessing construct summary scores coded scores above the average as 1 and scores below the average as 0.

^d^N/A = not applicable (n=1 in a cell of 2-by-2 table, p>0.99; and/or no convergence achieved)

**Appendix 7. Survey Initiation and Completion**

stratified by study arm

|  | **Study Arm, N (%)^a^** | | | |  |
| --- | --- | --- | --- | --- | --- |
|  | **Practice/Provider and Patient Interventions** | **Practice/Provider Intervention** and Patient Control | Practice/Provider Control and **Patient Intervention** | Practice/Provider and Patient Controls | Total |
| **Survey Initiation** |  |  |  |  |  |
| Baseline | 522 (100) | 523 (100) | 519 (100) | 523 (100) | 2087 (100) |
| P-value^b^ | 0.495 | | | | |
| One Month After Infant's Birth | 379 (73) | 404 (77) | 375 (72) | 399 (76) | 1557 (75) |
| P-value^b^ | 0.149 | | | | |
| One Year After Infant's Birth | 284 (54) | 285 (54) | 272 (52) | 294 (56) | 1135 (54) |
| P-value^b^ | 0.676 | | | | |
|  |  |  |  |  |  |
| **Survey Completion** |  |  |  |  |  |
| Baseline | 522 (100) | 523 (100) | 519 (100) | 522 (100) | 2086 (100) |
| P-value^b^ | 0.543 | | | | |
| One Month After Infant's Birth | 375 (72) | 394 (75) | 366 (71) | 389 (74) | 1,524 (73) |
| P-value^b^ | 0.269 | | | | |
| One Year After Infant's Birth | 279 (53) | 277 (53) | 270 (52) | 291 (56) | 1,117 (54) |
| P-value^b^ | 0.687 | | | | |

^a^Numbers in parentheses refer to column percentages (proportion of study participants in each arm initiating/completing each survey).

^b^P-value for the Pearson chi-squared proportion test at significance level of (a) 5%; bolded if significant.

**Appendix 8. Follow-Up Survey Completion**

stratified by maternal and infant vaccine intentions at baseline

|  | **Baseline Vaccine Intentions, N (%)** | | | | | |  |
| --- | --- | --- | --- | --- | --- | --- | --- |
|  | **Maternal Influenza Vaccine** | | **Maternal Tdap Vaccine** | | **All Infant Vaccines On Time** | |  |
|  | No/Unsure | Yes | No/Unsure | Yes | No/Unsure | Yes | Total |
| **Follow-up Survey** |  |  |  |  |  |  |  |
| One Month After Birth | 488 (64) | 1,036 (78) | 461 (64) | 1,063 (78) | 250 (63) | 1,271 (75) | 1,524 (73) |
| P-value^b^ | <0.001 | | <0.001 | | <0.001 | |  |
| One Year After Birth | 348 (46) | 769 (58) | 321 (45) | 796 (58) | 165 (41) | 951 (56) | 1,116 (54) |
| P-value^b^ | <0.001 | | <0.001 | | <0.001 | |  |

^a^Baseline survey questions assessing maternal and infant vaccine intentions, respectively, were: "Current guidelines suggest pregnant women to receive two vaccines while pregnant, flu and whooping cough. I intend to get: 1) both flu and whooping cough vaccines; 2) flu but not whooping cough vaccine; 3) whooping cough but not flu vaccine; 4) no vaccines; 5) not sure" and "Current guidelines suggest babies receive several vaccines. Regarding the vaccinations my doctor recommends for my baby after birth, I intend to get my baby: 1) all recommended vaccines on time; 2) all recommended vaccines but some spread out past the recommended ages; 3) some recommended vaccines but each on time; 4) some recommended vaccines spread out past the recommended ages; 5) no vaccines; 6) I'm not sure yet".

^b^P-value for the Pearson chi-squared proportion test at significance level of (a) 5%; bolded if significant.

**Appendix 9. Response Rates for Vaccine KAB Constructs at Each Study Timepoint**

|  | *Baseline*  *N (%)^b^* | *One Month After Birth*  *N (%)^b^* | *One Year After Birth*  *N (%)^b^* |
| --- | --- | --- | --- |
| **Total Initiating Survey** | 2087 | 1557 (100) | 1135 (100) |
|  |  |  |  |
| **KAB Constructs** ^a^ |  |  |  |
| Specific safety concerns (for infant vaccines) | 2082 (100) | 1523 (98) | 1124 (99) |
| Perceived risk (maternal influenza) | 1564 (75) | 1531 (98) | N/A^c^ |
| Confidence in vaccine efficacy (maternal influenza vaccine) | 1565 (75) | 1530 (98) | N/A^c^ |
| Perceived risk (infant whooping cough) | 1567 (75) | 1527 (98) | 1130 (100) |
| Confidence in vaccine efficacy (whooping cough vaccine) | 1567 (75) | 1531 (98) | N/A^c^ |
| Pro-vaccine social norms | 2083 (100) | 1524 (98) | 1128 (99) |
| Perceived vaccine knowledge | 2083 (100) | 1525 (98) | 1128 (99) |
| Trust in vaccine information (from obstetricians and pediatricians) | 2084 (100) | 1525 (98) | 1128 (99) |
| Trust in vaccine information (from naturopaths and chiropractors) | 1358 (65) | 834 (55) | 587 (52) |
| Trust in vaccine information (from federal agencies and academic institutions) | 2084 (100) | 1524 (98) | 1128 (99) |

^a^KAB = knowledge, attitudes, and beliefs. Summary scores were created for all KAB constructs with multiple constituent survey statements at each timepoint (see Appendix 1).

^b^Numbers in parentheses refer to column percentages (response rate for the survey statements of each KAB construct among those initiating each survey).

^c^N/A = not applicable (this KAB construct was not assessed at this timepoint)

**Appendix 10. Maternal and Infant Vaccine Intentions at Baseline**

stratified by 4 study arms

|  | **Study Arm, N (%)** | | | |  |
| --- | --- | --- | --- | --- | --- |
|  | **Practice/Provider and Patient Interventions** | **Practice/Provider Intervention** and Patient Control | Practice/Provider Control and **Patient Intervention** | Practice/Provider and Patient Controls | Total |
| **Maternal Vaccine Intentions**^a^ |  |  |  |  |  |
| Influenza and Tdap | 292 (56) | 290 (55) | 286 (55) | 311 (59) | 1179 (56) |
| Influenza not Tdap | 45 (9) | 35 (7) | 36 (7) | 33 (6) | 149 (7) |
| Tdap not Influenza | 45 (9) | 45 (9) | 56 (11) | 45 (9) | 191 (9) |
| Neither | 64 (12) | 83 (16) | 82 (16) | 73 (14) | 302 (14) |
| Unsure | 76 (15) | 70 (13) | 59 (11) | 61 (12) | 266 (13) |
| Total | 522 (100) | 523 (100) | 519 (100) | 523 (100) | 2087 (100) |
| P-value^b^ | 0.495 | | | | |
|  |  |  |  |  |  |
| **Infant Vaccine Intentions**^a^ |  |  |  |  |  |
| All On Time | 357 (69) | 349 (67) | 351 (68) | 368 (70) | 1425 (68) |
| All But Delayed | 66 (13) | 66 (13) | 74 (14) | 54 (10) | 260 (12) |
| Some But On Time | 28 (5) | 37 (7) | 24 (5) | 27 (5) | 116 (6) |
| Some But Delayed | 17 (3) | 14 (3) | 14 (3) | 18 (3) | 63 (3) |
| None | 12 (2) | 12 (2) | 8 (2) | 10 (2) | 42 (2) |
| Unsure | 41 (8) | 44 (8) | 47 (9) | 45 (9) | 177 (8) |
| Total | 521 (100) | 522 (100) | 518 (100) | 522 (100) | 2083 (100) |
| P-value^b^ | 0.864 | | | | |

^a^Baseline survey questions assessing maternal and infant vaccine intentions, respectively, were: "Current guidelines suggest pregnant women to receive two vaccines while pregnant, flu and whooping cough. I intend to get: 1) both flu and whooping cough vaccines; 2) flu but not whooping cough vaccine; 3) whooping cough but not flu vaccine; 4) no vaccines; 5) not sure" and "Current guidelines suggest babies receive several vaccines. Regarding the vaccinations my doctor recommends for my baby after birth, I intend to get my baby: 1) all recommended vaccines on time; 2) all recommended vaccines but some spread out past the recommended ages; 3) some recommended vaccines but each on time; 4) some recommended vaccines spread out past the recommended ages; 5) no vaccines; 6) I'm not sure yet".

^b^P-value for the Pearson chi-squared proportion test at significance level of (a) 5%; bolded if significant.
